# Unveiling the Immunopharmacological Mechanisms of Biejiajian Pills (BJJP) Underlying the Treatment of Hepatic Fibrosis: Insights From Network Pharmacology and Experimental Validation

**DOI:** 10.1002/fsn3.71683

**Published:** 2026-03-23

**Authors:** Chan Mo, Zhuolin Wei, Jinnan He, Yuan Liu, Jiaorong Zheng, Min Hong, Yuhong Song

**Affiliations:** ^1^ Department of First Clinical Medical College Guangdong Pharmaceutical University Guangzhou Guangdong PR China; ^2^ College of Integrated Traditional Chinese and Western Medicine Jining Medical University Jining Shandong PR China; ^3^ School of Traditional Chinese Medicine Guangdong Pharmaceutical University Guangzhou Guangdong PR China

**Keywords:** Biejiajian Pills (BJJP), hepatic fibrosis (HF), immune infiltration, network pharmacology, UHPLC‐HRMS

## Abstract

Biejiajian Pills (BJJP) have demonstrated certain therapeutic effects in the treatment of hepatic fibrosis (HF) in Traditional Chinese medicine practice. However, their underlying mechanism of action remains unclear. This study aimed to investigate the primary compounds of BJJP and their potential mechanisms in treating HF through network pharmacology and experimental validation. By integrating network pharmacology with RF, SVM‐RFE, and LASSO algorithms, this study identified that the therapeutic effect of BJJP on HF may be mediated by two signature genes, LYN and CFTR, which were further validated using a validation set. Molecular docking results showed that the main compounds of BJJP, including six compounds such as kaempferol and baicalein, have a strong binding affinity with LYN and CFTR. Through the ssGSEA algorithm, we found that the proportions of six immune cell subsets, including M1 macrophages, M2 macrophages, and resting memory CD4^+^T cells, showed directional changes between the HF group and the control group, and these changes were significantly correlated with the expression of LYN and CFTR. Furthermore, 133 compounds in BJJP were characterized using UHPLC‐HRMS. In vivo experiments confirmed that BJJP effectively ameliorated liver function–related biochemical indices and histopathological alterations in mice with HF, downregulated the expression levels of LYN and CFTR, reduced the infiltration of CD45^+^, CD8^+^, CD68^+^, and TNF‐α^+^ cells, and elevated the proportions of CD4^+^ and CD163^+^ cells. Bioinformatics analyses combined with experimental validation have demonstrated that BJJP exerts therapeutic effects on HF by downregulating the expression of LYN and CFTR and modulating immune function.

## Graphical abstract

This study employed an integrated workflow combining network pharmacology, machine learning, molecular docking, UHPLC‐HRMS, and in vivo validation. The core design aimed to identify key compounds and signature genes of BJJP against HF, explore their immune‐related mechanisms, and verify the therapeutic targets and pathways through experimental validation.

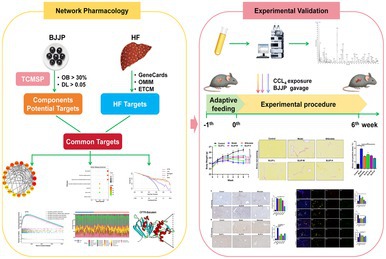

**Abbreviations**
ALT: alanine aminotransferase
AST: aspartate aminotransferase
AUC: area under the curve
BJJP: Biejiajian Pill
BMSCs: bone mesenchymal stem cells
BP: biological process
CC: cellular components
CFTR: cystic fibrosis transmembrane conductance regulator
CSCs: cancer stem cells
DEGs: differentially expressed genes
DL: drug‐likeness
ECM: extracellular matrix
GBD: Global Burden of Disease
GEO: Gene Expression Omnibus
GSEA: Gene Set Enrichment Analysis
HCC: hepatocellular carcinoma
HF: hepatic fibrosis
HSCs: hepatic stellate cells
LYN: Lck/Yes‐related novel protein tyrosine kinase
NAFLD: non‐alcoholic fatty liver disease
NASH: non‐alcoholic steatohepatitis
OB: oral bioavailability
ROC: receiver operating characteristic
TCM: Traditional Chinese medicine
TF: transcription factor
UHPLC‐HRMS: ultra‐high‐performance liquid chromatography‐high‐resolution mass spectrometry
α‐SMA: α‐smooth muscle actin

## 1 Introduction

Hepatic fibrosis (HF), a common pathological sequela of chronic hepatopathies, involves excessive hepatocellular hyperplasia, architectural remodeling of liver parenchyma, fibrous tissue expansion, and pseudolobule formation upon hepatic insult (Lan et al. 2025). Its primary etiologies include non‐alcoholic fatty liver disease (NAFLD), non‐alcoholic steatohepatitis (NASH), viral hepatitis, and alcohol‐associated liver disease, with the global prevalence of NAFLD and NASH escalating substantially to become pivotal drivers in HF (Hammerich and Tacke 2023). HF imposes a growing global burden: the 2020 Global Burden of Disease (GBD) study estimated 112 million global cases of compensated liver cirrhosis, while a 2017–2022 Chinese nationwide study reported a 2.85% prevalence of liver fibrosis among adults (Danpanichkul et al. 2025; Sun and Guo 2025; Tang et al. 2025). HF accompanies the progression of chronic liver disease regardless of its etiology. Its therapeutic goal is to slow or reverse progression and prevent progression to cirrhosis or hepatocellular carcinoma (HCC), as reversal of advanced fibrosis or cirrhosis is slow and often unachievable. At present, there is no specific drug for HF that has been approved by the FDA, while some agents have shown anti‐HF potential in clinical trials, their efficacy remains suboptimal (Zhang et al. 2023), highlighting the urgency of investigating HF mechanisms and identifying potential biomarkers for early diagnosis and treatment.

Traditional Chinese medicine (TCM) has gained increasing attention for its unique merits in HF management, with extensive clinical and preclinical evidence supporting the anti‐fibrotic potential of TCM herbal preparations (Luo et al. 2025). Representative formulations such as Biejia‐Ruanggan Tablets (Meng et al. 2022; Xu and Xue 2022), Anluo Huaxian Pills (Xiao et al. 2022), and Fuzheng Huayu Formula (Hassanein et al. 2022) have been shown to markedly alleviate clinical symptoms in patients with HF and effectively retard its progression. Experimental studies suggest that the mechanisms underlying TCM's anti‐fibrotic action are complex and multitargeted, involving the inhibition of hepatic stellate cell (HSC) activation and proliferation (Boeff et al. 2025), attenuation of lipid peroxidation‐induced damage, modulation of extracellular matrix (ECM) synthesis and degradation, and regulation of immune homeostasis to mitigate liver inflammatory responses.

Biejiajian Pills (BJJP), derived from the classic medical text Essential Prescriptions from the Golden Chamber (Luo et al. 2025), are formulated with 23 herbal components (see Table S1 for details) (Chen et al. 2023). According to TCM theory, HF primarily results from the accumulation of dampness‐heat and toxins, compounded by qi deficiency due to irregular habits. This leads to internal organ dysfunction, blood stasis in the liver and spleen, and obstruction of meridians. Consequently, the therapeutic principles for HF emphasize activating blood circulation to dissipate stasis, strengthening the spleen, soothing the liver, and regulating qi. BJJP aligns with these principles, featuring a formulation that simultaneously addresses phlegm and blood stasis through the integration of cold and heat properties, purgative and tonic effects. Its broad therapeutic efficacy has been demonstrated in the treatment of HF (Cheng et al. 2023), liver cirrhosis (Chi et al. 2023), NAFLD (Huang et al. 2024), and hyperlipidemia (Zhu et al. 2022). Experimental evidence suggests that BJJP exerts therapeutic effects through diverse mechanisms. In oncology studies, the synergistic action of BJJP and bone mesenchymal stem cells (BMSCs) was found to enhance the regulation of cancer stem cells (CSCs) by modulating miR‐140 and inhibiting the Wnt/β‐catenin pathway, thereby suppressing HCC (Jingjing et al. 2021, 2022). Additionally, BJJP inhibits HCC progression by downregulating the PDGFRβ pathway in cancer‐associated fibroblasts (CAFs), consequently suppressing VEGF‐A and HGF expression (Chen, He, et al. 2025). Liu et al. revealed that BJJP ameliorated BDL‐induced cholestatic HF in rats by regulating intestinal microbiota and the TMAO‐mediated PI3K/AKT signaling pathway (Cui et al. 2025). Clinically, BJJP effectively reduces liver stiffness measurement (LSM) values and HF biomarkers, including ECM components (HA, LN, PC‐III, IV‐C), demonstrating significant efficacy in improving or reversing HF without obvious side effects (Cheng et al. 2023; Chi et al. 2023; Zhang, Yang, et al. 2019). Consequently, the 2023 edition of the *Guidelines for the Diagnosis and Treatment of Liver Fibrosis with Integrated Traditional Chinese and Western Medicine* designates BJJP as an effective therapeutic formulation for HF (Lyu et al. 2025). Despite these advances and the verified safety profile of BJJP, the precise molecular mechanisms underlying its anti‐fibrotic effects remain incompletely elucidated.

Network pharmacology is an interdisciplinary approach that integrates systems biology and bioinformatics to elucidate the complex, multi‐component mechanisms of drugs by analyzing interconnected “drug‐target‐disease” networks (Hopkins 2008; Li et al. 2023; Zhao and Iyengar 2012). In recent years, the integration of machine learning algorithms, such as the Least Absolute Shrinkage and Selection Operator (LASSO), Random Forest (RF), and Support Vector Machine‐Recursive Feature Elimination (SVM‐RFE), has significantly enhanced the precision of core target identification in TCM, effectively addressing the “multi‐target, multi‐pathway” complexity of Chinese herbal medicines (Liang, Liu, et al. 2025; Liang, Ning, et al. 2025; Meng et al. 2026; Ye et al. 2025). Furthermore, the single‐sample Gene Set Enrichment Analysis (ssGSEA) has emerged as a powerful tool for characterizing immune infiltration patterns in liver fibrosis, providing critical insights into the immunomodulatory effects of TCM (Xie, Xie, et al. 2022; Yang et al. 2025). Building on these advanced analytical platforms, in this study, we employed network pharmacology to identify key compounds and molecular targets of BJJP in HF, followed by functional enrichment analysis. Specifically, we utilized three machine learning algorithms (LASSO, RF, and SVM‐RFE) to screen core target genes, while predicting associated miRNAs and TFs. Immune infiltration profiles were investigated using ssGSEA, and molecular docking was performed to verify compound‐target interactions. Furthermore, we identified BJJP compounds via UHPLC‐HRMS and validated its therapeutic efficacy and key targets in a CCl_4_‐induced mouse model of HF. This study establishes a comprehensive framework for investigating the pharmacological effects of BJJP, laying a solid foundation for future research into its in vivo mechanisms.

## 2 Materials and Methods

### 2.1 Data Acquisition

The NCBI Gene Expression Omnibus (GEO, https://www.ncbi.nlm.nih.gov/geo/) is a public database that archives and freely distributes high‐throughput gene expression data, including microarray and next‐generation sequencing datasets (Barrett et al. 2013). The inclusion criteria for dataset selection used in this study were as follows: (1) The dataset contains independent gene expression profiles of patients with HF; (2) The dataset includes information on both HF cases and normal control (Control) subjects; (3) The test specimens in the dataset are derived from human tissues. After screening, the original data for this study were downloaded from GEO as follows: the dataset GSE84044 (raw file: “GSE84044_RAW.tar”) was designated as the training set, and GSE171294 (raw file: “GSE171294_gene_fpmk.txt.gz”) served as the validation set. Raw data processing was performed using the R package affy (Version 1.74.0; https://bioconductor.org/packages/release/bioc/html/affy.html), which included background correction and normalization of the expression matrices, followed by gene annotation. Specifically, mRNA probe expression matrix files for each dataset were downloaded, along with the corresponding annotation files of their respective sequencing platforms. Probes were individually converted to gene symbols, with those failing to map to any gene symbol excluded. For multiple probes mapping to the same gene, the mean value was calculated as the representative expression level of that gene. This process yielded the gene expression matrices used for subsequent analyses.

### 2.2 Acquisition of BJJP‐Related Active Compounds and Target Genes

Active compounds of BJJP were primarily screened from the Traditional Chinese Medicine Systems Pharmacology Database (TCMSP) using the criteria of oral bioavailability (OB) > 30% and drug‐likeness (DL) > 0.18, and were further supplemented with candidate compounds from the Encyclopedia of Traditional Chinese Medicine (ETCM). Target genes corresponding to these active compounds were predicted using TCMSP and SwissTargetPrediction (prediction probability > 0). The obtained gene symbols were standardized according to HGNC nomenclature, followed by removal of duplicate entries and exclusion of non‐human targets. Detailed information on compound screening and target prediction is provided in Supporting Information Method S1.

### 2.3 Construction of a Drug‐Target Atlas

BJJP, as a composite herbal formula, consists of 23 herbal components (see Table S1 for details). All potential targets and their corresponding active compounds of the 23 herbal components in BJJP were integrated. The integrated data were imported into Cytoscape (Version 3.10.2) to construct a “TCM‐Compound‐Target” network, visually presenting the associations between BJJP's herbal ingredients, active compounds, and predicted target genes.

### 2.4 Acquisition of HF‐Related Targets and Analysis by Multiple HF‐Related Databases

HF‐related target genes were retrieved from GeneCards (Relevance score > 1, Protein Coding), OMIM, and ETCM. After unifying gene symbols (HGNC nomenclature), removing duplicates and excluding uncharacterized transcripts, non‐redundant HF‐related target genes were obtained. Differential expression analysis between the HF group and normal control group was performed using the R package limma (Version 3.52.4), with differentially expressed genes (DEGs) screened by log_2_FC > 0.263 and adj. *p*‐value < 0.05. Detailed procedures for gene retrieval and DEG analysis are described in Supporting Information Method S2.

### 2.5 Construction and Enrichment Analysis of Protein–Protein Interaction (PPI) Networks for Drug‐Target Genes

The STRING database (Version 12.0, https://cn.string‐db.org/) was used to retrieve PPI relationships among HF‐related gene products. A PPI network was constructed with a threshold of combined score > 0.15. For the screened drug‐target genes, GO functional enrichment and KEGG pathway enrichment analyses were performed using the R package clusterProfiler (Version 4.10.0; http://bioconductor.org/packages/release/bioc/html/clusterProfiler.html) to explore the biological functions and key pathways involved in these genes. Multiple test correction was conducted using the Benjamini & Hochberg method, yielding adjusted *p*‐values (adj. *p*‐value). The screening thresholds were set as follows: adj. *p*‐value < 0.05 for both GO and KEGG analyses. Finally, the top five most significant GO enrichment results and top 10 most significant KEGG enrichment results were visualized.

### 2.6 Identify the Characteristic Genes of HF Lesions

Three machine learning algorithms (LASSO, RF, SVM‐RFE) were employed to screen characteristic genes from the common targets of BJJP and HF. LASSO regression was used for dimensionality reduction and feature selection via 10‐fold cross‐validation; RF was applied to rank gene importance based on MeanDecreaseGini (threshold: > 0.25); SVM‐RFE was utilized for recursive feature elimination and validation via 10‐fold cross‐validation. Genes intersecting among the three algorithms were defined as core characteristic genes of HF. Detailed parameters and implementation procedures are provided in Supporting Information Method S3.

### 2.7 Verification and Performance Evaluation of Characteristic Genes

Using data from both the training set and validation set, receiver operating characteristic (ROC) curves and area under the curve (AUC) values were plotted and calculated across all datasets (including the training and validation sets) via the R package pROC (Version 1.18.5, http://expasy.org/tools/pROC/) (Robin et al. 2011) to assess the diagnostic accuracy of the characteristic genes. Diagnostic genes with an AUC > 0.7 were retained for subsequent analyses.

### 2.8 Gene Set Enrichment Analysis (GSEA)

GSEA is a computational method used to evaluate whether a group of genes exhibits statistically significant differences between two biological states (Reimand et al. 2019). In this study, GSEA was employed to analyze the enrichment of the identified characteristic genes. The “h.all.v7.5.1.symbols” gene set was downloaded from the Molecular Signatures Database (MSigDB; http://www.gsea‐msigdb.org), and gene set enrichment analysis was conducted using this dataset. The statistical significance threshold was set at a false discovery rate (FDR) < 0.05.

### 2.9 Immunological Characteristics of HF

Single‐sample gene set enrichment analysis (ssGSEA), an extension of the GSEA method, is widely applied in bioinformatics studies focusing on immune infiltration. In this study, the enrichment scores of 22 immune cell types in both normal and HF samples were calculated using the R package GSVA. Additionally, Spearman correlation analysis was performed to assess the correlations between Hub HF‐DEGs and immune cells.

### 2.10 Molecular Docking

Molecular docking was performed to verify the binding potential between the key active compounds of BJJP and the core characteristic gene‐encoded proteins (CFTR and LYN). The crystal structures of target proteins were retrieved from the Protein Data Bank (PDB), with specific structures selected based on functional domains. Detailed procedures are provided in Supporting Information Method S4.

### 2.11 Characterization of Primary Chemical Constituents of BJJP by UHPLC‐HRMS

Two grams of BJJP (Sinopharm Group Zhonglian Pharmaceutical Co. Ltd.) were weighed, crushed, and mixed with 40 mL of 80% aqueous methanol. Ultrasonic extraction was performed for 30 min, after which 1 mL of the BJJP test sample was accurately withdrawn, thoroughly mixed, and filtered through a 0.22‐μm filter membrane. The resulting filtrate was used as the test solution. The instrument parameters and detailed testing conditions were presented in Supporting Information Method S5.

### 2.12 Experimental Animals

Male C57BL/6J mice, weighing 20 ± 2 g, were of specific pathogen‐free (SPF) grade and purchased from Guangdong Yaokang Biotechnology Co. Ltd. Before the experiment, the animals underwent a 7‐day adaptation period, during which standard laboratory feed and water were provided. The ambient temperature was maintained at 20°C–26°C, and the light/darkness cycle was 12 h. All animal experimental protocols complied with the National Guidelines for the Management and Use of Laboratory Animals and had been approved by the Laboratory Animal Ethics Review Committee of Shenzhen Zhongxun Precision Medicine Research Institute (Animal Ethics No: ZXJZ202401010008). Humane endpoint criteria were strictly followed throughout the experiment. Mice were humanely euthanized if they met any of the following conditions: (1) body weight loss exceeding 20% of initial body weight; (2) severe lethargy, inability to access food or water, or persistent hunched posture for more than 24 h; and (3) visible signs of severe pain or distress, such as persistent vocalization or self‐mutilation. Euthanasia was performed by cervical dislocation after deep anesthesia with isoflurane inhalation, and all efforts were made to minimize animal suffering.

After the adaptation period ended, the mice were randomly divided into six groups: control group, HF group, positive control drug group (Bifendate, 2.92 mg/kg), low‐dose BJJP group (BJJP‐L, 1.18 g/kg), medium‐dose BJJP group (BJJP‐M, 2.36 g/kg), and high‐dose BJJP group (BJJP‐H, 4.72 g/kg). Except for the control group, all groups in the HF model were administered an intraperitoneal injection of 25% CCl_4_‐olive oil solution (CCl_4_: olive oil = 1:3) at a dose of 2 mL/kg·d. Mice in the control group were intraperitoneally injected with pure olive oil at a dose of 1.5 mL/kg·d, three times a week for 6 weeks. Meanwhile, starting from the initiation of the injection, mice in each treatment group were given the corresponding test drug by gavage at a volume of 20 mL/kg per day, whereas the control group and HF group were gavaged with the same volume of normal saline for 6 weeks.

### 2.13 Detection of AST and ALT in Mouse Serum

According to the manufacturer's instructions (Nanjing Jiancheng Bioengineering Institute), an appropriate amount of pretreated serum sample was incubated with the corresponding reagents under specified conditions (37°C). Absorbance was measured using a multifunctional microplate reader (TECAN Spark, Switzerland). The activities of AST and ALT were then calculated based on the standard curve.

### 2.14 H&E and Sirius Red Staining

H&E staining and Sirius Red staining were performed on mouse liver tissue sections to observe histopathological changes and collagen deposition, respectively. The detailed procedures of both staining methods, including tissue fixation, dehydration, embedding, sectioning, dewaxing, staining, dehydration, clearing and mounting, are described in detail in Supporting Information Method S6.

### 2.15 Immunohistochemical Staining

Immunohistochemical staining was performed on mouse liver tissue sections to detect the expression levels of LYN and CFTR proteins. The detailed procedural steps of the staining experiment, including dewaxing, antigen retrieval, blocking, antibody incubation, chromogenic reaction, and mounting, were described in detail in Supporting Information Method S7.

### 2.16 Immunofluorescence Staining

Immunofluorescence staining was performed on mouse liver tissue paraffin sections to detect the expression of target proteins. α‐SMA immunofluorescence staining was conducted to observe α‐SMA expression, and fluorescence four‐marker staining was carried out to detect the expressions of CD45, CD4, CD8, CD163, CD68, and TNF‐α. The detailed procedural steps of both staining methods, including dewaxing, antigen retrieval, blocking, antibody incubation, staining, mounting, and image collection, are described in detail in Supporting Information Method S8.

### 2.17 Statistical Analysis

Data were analyzed using SPSS 23.0 software, and graphs were generated using GraphPad Prism 9.0. Quantitative data were expressed as mean ± standard error of the mean (SEM). One‐way analysis of variance (ANOVA) was applied for comparisons among multiple groups. For pairwise comparisons, the Tukey test was used when variances were homogeneous, whereas Dunnett's T3 test was employed when variances were heterogeneous. A *p*‐value < 0.05 was considered statistically significant.

## 3 Results

### 3.1 Screening of BJJP Compound Targets and HF Disease Targets

**FIGURE 1:**
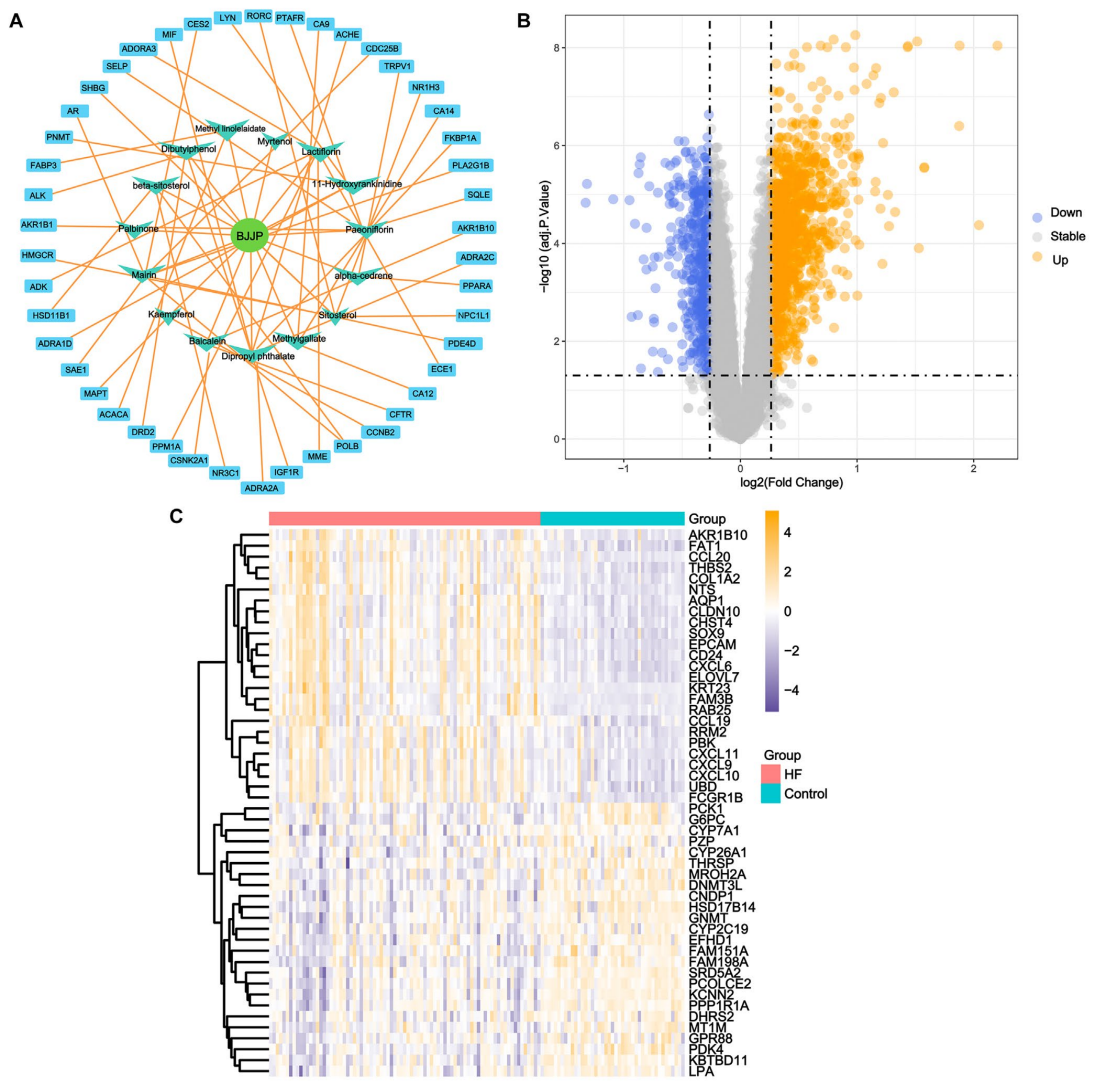
Screening of BJJP compound targets and HF disease targets. (A) Active compound‐target network diagram of BJJP. (B) Volcano plot of DEGs, where blue indicates downregulated genes and yellow indicates upregulated genes. (C) Heatmap of the top 20 upregulated and downregulated differentially expressed genes. BJJP, Biejiajian Pills; DEGs, differentially expressed genes; HF, hepatic fibrosis.

A total of 1009 target genes were collected after merging and deduplication of targets corresponding to all compounds in BJJP, while 1025 TCM‐related targets were obtained via target prediction using the TCMSP database alone. A partial network map was constructed and is presented in Figure 1A. The GeneCards (www.genecards.org) database yielded 1024 liver fibrosis targets, the OMIM database collected 97 targets, and the ETCM database retrieved 72 targets. After merging and removing duplicates, a total of 1046 related genes were obtained. Using the method described, differential analysis of the training set was performed with limma, as shown in Figure 1B, resulting in 1592 DEGs, among which 1087 were upregulated and 505 were downregulated. The heat map of the top 20 upregulated and downregulated gene expressions is shown in Figure 1C.

### 3.2 The PPI Network of BJJP and HF Common Targets

**FIGURE 2:**
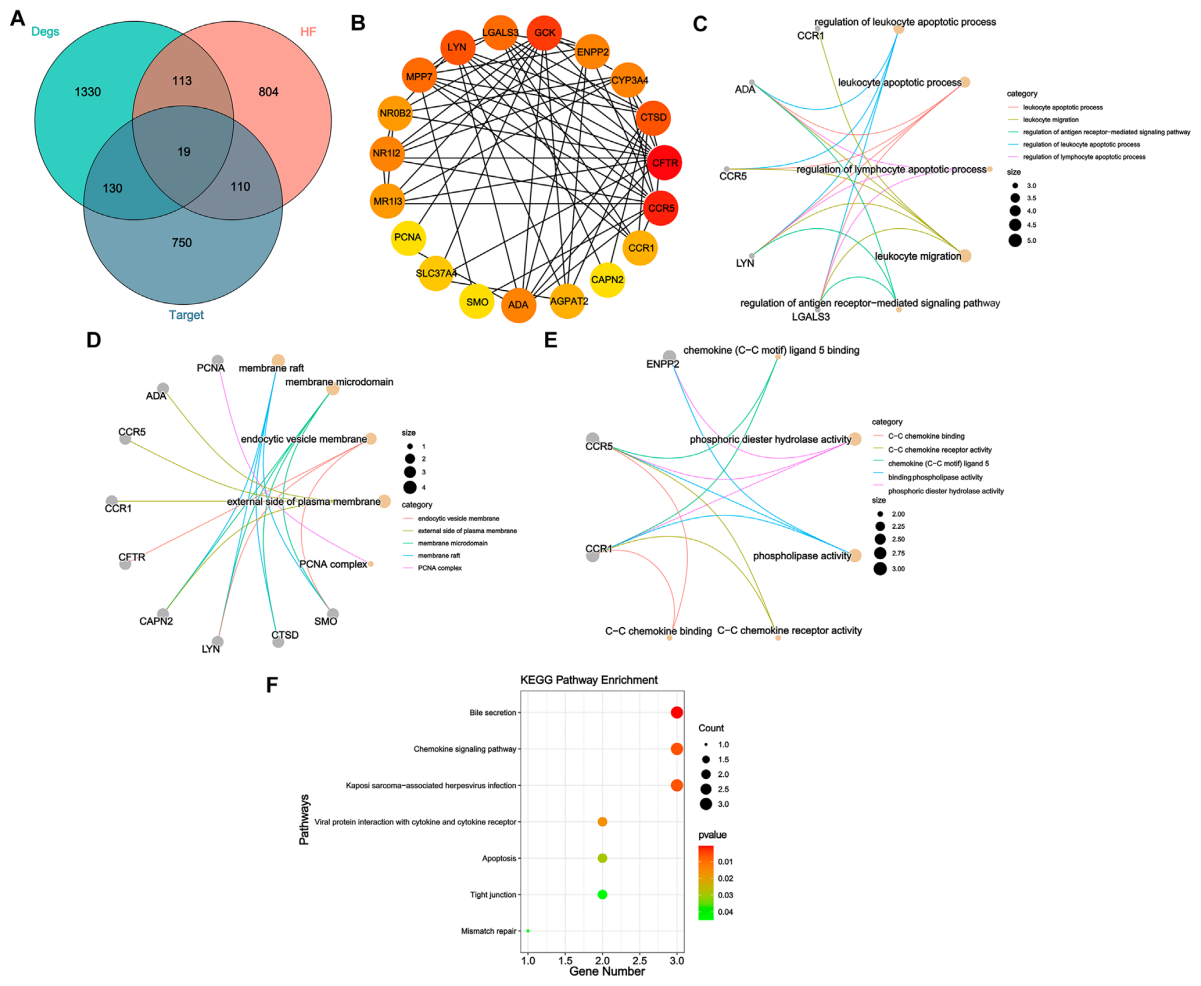
The PPI network of BJJP and HF common targets. (A) Venn diagram of the intersection among DEGs, drug targets, and HF‐related genes. (B) The PPI network of 19 targets according to the STRING database. (C) GO enrichment analysis with BP, CC (D), and MF (E) included. (F) KEGG enrichment analysis bubble plot. BP, biological process; CC, cellular components; MF, molecular function; PPI, protein–protein interactions.

Nineteen genes were obtained by intersecting DEGs, drug targets, and HF‐related genes, and were visualized using a Venn diagram (Figure 2A). The above‐mentioned 19 drug‐target genes were imported into the STRING database to construct a PPI network, as shown in Figure 2B. Subsequently, GO functional annotation and KEGG signaling pathway enrichment analyses were performed on these 19 drug‐target genes to explore the functional terms involved in the key genes. The enrichment results were shown in Figure 2C–F (sorted by ascending P‐value). With a threshold of *p* < 0.05, significantly enriched pathways were identified. GO enrichment results indicated that, at the biological process level, the genes were significantly enriched in biological processes including “regulation of leukocyte apoptotic process” and “leukocyte apoptotic process”. At the cellular component level, the genes were enriched in membrane‐related structures such as “membrane raft” and “membrane microdomain.” At the molecular function level, these genes were mainly associated with “phosphoric diester hydrolase activity” and “chemokine (C–C motif) ligand 5 binding.” The KEGG enrichment results indicated that the drug‐target genes were significantly enriched in pathways including “Bile secretion” and “Chemokine signaling pathway.”

### 3.3 Identification Characteristic Genes Related to HF

**FIGURE 3:**
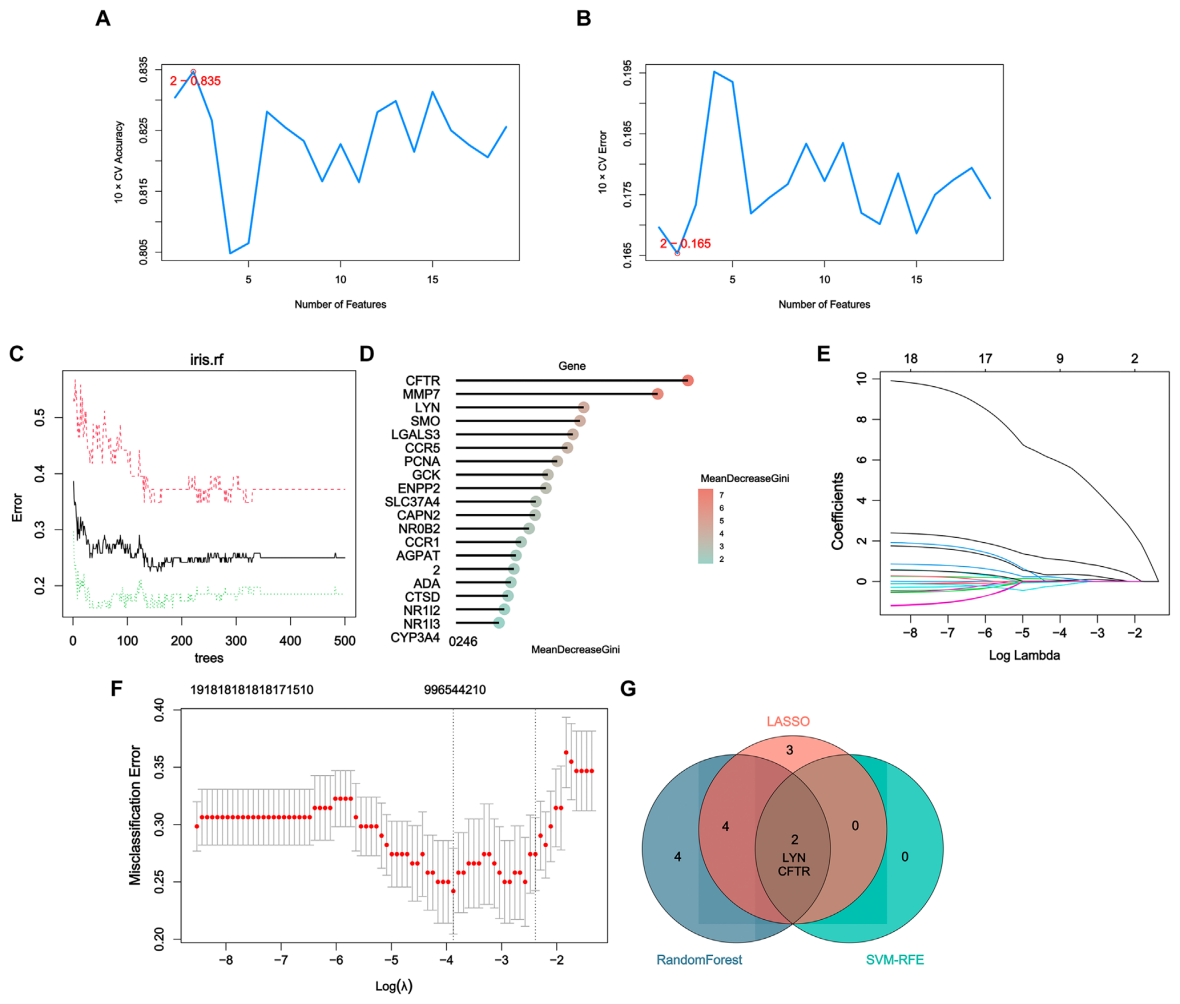
Identification characteristic genes related to HF. (A) Graph of the relationship between error rate and number of features in the SVM‐RFE feature selection algorithm. (B) Graph of the relationship between accuracy and number of features in the SVM‐RFE feature selection algorithm. (C) Graph of the relationship between the number of trees and error rate in RF. (D) Arrange according to the relative importance of genes. (E) Ten cross‐validations for optimizing parameter selection in the LASSO model. Each curve corresponds to a gene. (F) LASSO coefficient profile. Solid vertical lines represent partial likelihood bias SE. (G) Venn diagram of signature genes shared among LASSO, RF, and SVM‐RFE algorithms.

Two algorithms were applied to select feature genes from the 19 target genes. For the SVM‐RFE algorithm, when the number of features was 2, the classifier error was the lowest, with the results shown in Figure 3A,B. The random forest algorithm selected the top 10 genes ranked by MeanDecreaseGini, and the results were presented in Figure 3C,D. For the LASSO algorithm, after 10‐fold cross‐validation, the combination with minimum error and highest accuracy was chosen to construct the LASSO classifier, and a total of nine feature genes were identified (Figure 3E,F). This classifier had the smallest error. After taking the intersection, two characteristic genes, LYN and CFTR, common to the random forest, SVM‐RFE, and LASSO algorithms were finally identified, with the results shown in Figure 3G.

### 3.4 Validation Characteristic Genes Related to HF

**FIGURE 4:**
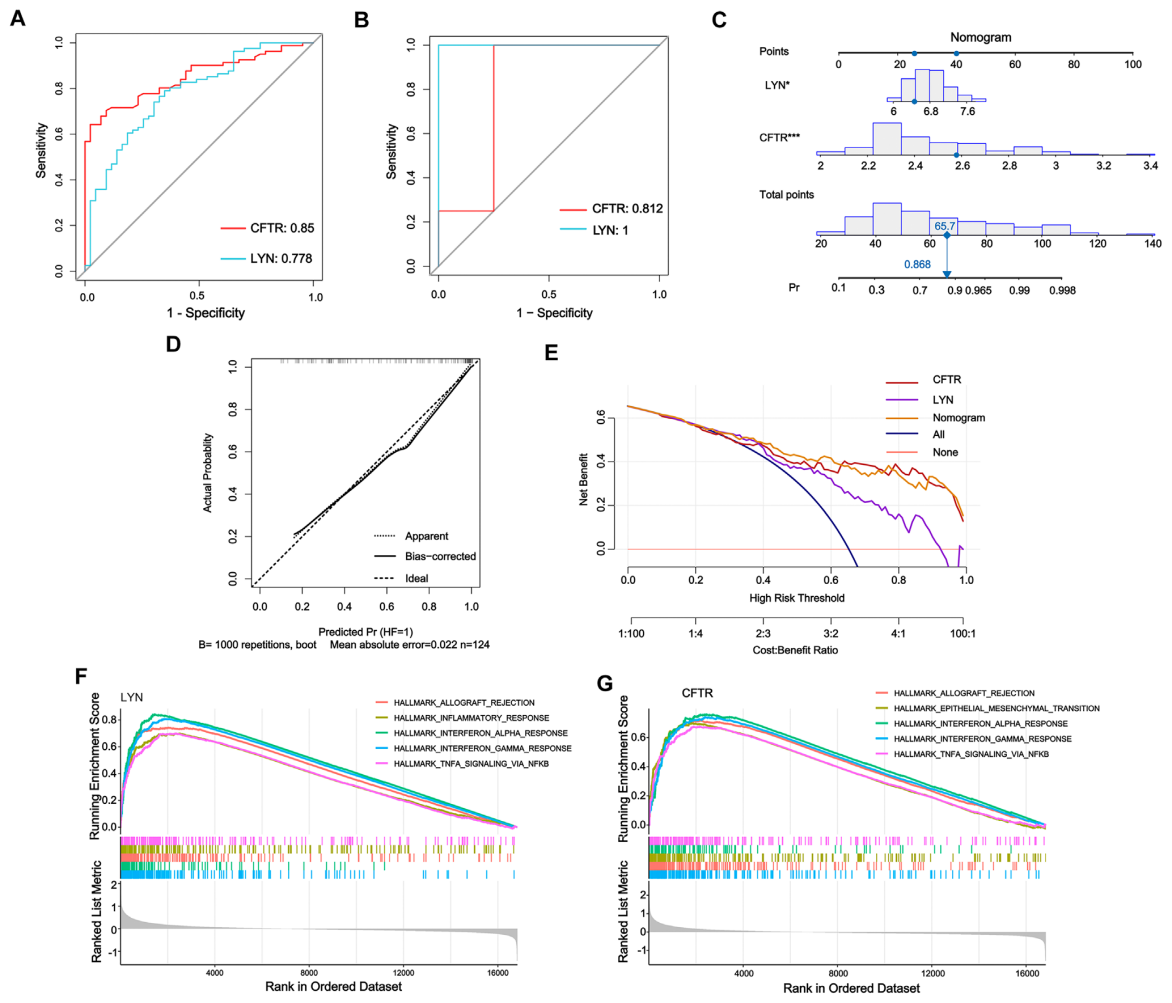
Validation characteristic genes related to HF. (A) The ROC curves of CFTR and LYN in the training set between the HF group and the normal control group. (B) The ROC curves of CFTR and LYN in the validation set between the HF group and the normal control group. (C) Nomogram of the risk model. (D) Calibration curve. (E) DCA curve. (F) GSEA visualization plot of LYN gene in Hallmark pathways. (G) GSEA visualization plot of CFTR gene in Hallmark pathways. CFTR: cystic fibrosis transmembrane conductance regulator; LYN: Lck/Yes‐related novel protein tyrosine kinase.

According to the analysis, the AUC values of CFTR and LYN in the training set (Figure 4A) and validation set (Figure 4B) between the HF group and the normal control group were both greater than 0.7. Subsequently, LYN and CFTR were subjected to further analysis. To verify the combined diagnostic value of LYN and CFTR for HF, these two genes were incorporated into the nomogram. The decision curve and calibration curve indicated that the nomogram exhibited good predictive performance (Figure 4C–E). We investigated the HALLMARKS pathway enrichment of LYN and CFTR in HF by GSEA, and the top five most significant pathways were obtained as shown in Figure 4F,G. From the enrichment results, it could be seen that LYN might be associated with “Allograft rejection” and “Inflammatory response,” and related to “IFN‐α response” and “IFN‐γ response,” suggesting LYN might play an important role in the physiological/pathological processes related to immune inflammation in HF and could serve as a direction for subsequent mechanistic research and target exploration. CFTR might be associated with “Allograft rejection” and “Epithelial–mesenchymal‐transition (EMT),” and related to “IFN‐α response,” “IFN‐γ response,” and “TNF‐α signaling/NF‐κB,” suggested that CFTR might play a key role in physiological/pathological processes, including immune responses in HF and tissue remodeling (such as fibrosis associated with EMT etc.).

### 3.5 Constructing the Regulatory Network of BJJP Against HF

We further constructed an association map titled “Core Active Compounds of BJJP‐Key Targets‐Disease Pathways” to systematically deduce the potential active compounds, functional targets, and their mechanistic links to involved signaling pathways underlying BJJP's therapeutic effects on HF. As illustrated in Figure S1A, baicalein, kaempferol, and luteolin directly target CFTR, a key regulator of the “Bile secretion” pathway. This indicates that these compounds may modulate bile secretion and hepatic‐biliary homeostasis by targeting CFTR, with implications for processes like substance transport and liver‐gallbladder pathophysiology. For the LYN‐mediated axis, anhydrobelachinal, tetrandrine, and 11‐hydroxyrankinidine act on LYN, which participates in both the “Chemokine signaling pathway” and “Kaposi sarcoma‐associated herpesvirus infection” pathway. This suggests that these compounds can interfere with immune cell trafficking, inflammatory responses, and viral infection‐related processes via LYN, potentially contributing to anti‐inflammatory, antiviral, or tumor‐suppressive regulatory cascades in HF. We utilized Cytoscape for visualization and constructed the “TF‐miRNA‐mRNA” network, as shown in Figure S1B. By integrating data from the miRTarBase and ChEA3 databases, this study predicted that CFTR and LYN might have regulatory relationships with 47 miRNAs and 7 transcription factors (TFs).

### 3.6 Molecular Docking Results Between the Components of BJJP Compound and the Target Protein

**FIGURE 5:**
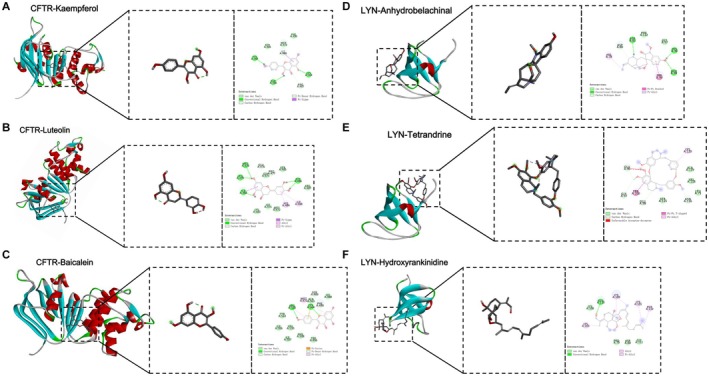
Molecular docking of BJJP active compounds with target proteins. (A) Kaempferol docking with CFTR. (B) Luteolin docking with CFTR. (C) Baicalein docking with CFTR. (D) Anhydrobelachinal docking with LYN. (E) Tetrandrine docking with LYN. (F) 11‐Hydroxyrankinidine docking with LYN.

To validate the results of network pharmacology, we employed molecular docking to verify the binding affinity between the key compounds of BJJP and CFTR, as well as LYN. Existing literature has demonstrated that a binding affinity of less than −4.25 kcal/mol indicates that standard binding between the two molecules; less than −5.0 kcal/mol indicates good binding; and less than −7.0 kcal/mol indicates strong binding activity (Xiang et al. 2022). We performed molecular docking between CFTR/LYN and their respective active compounds from BJJP, with the detailed results presented in Figure 5. The binding energies between CFTR and kaempferol, luteolin, and baicalein were −4.69, −5.52, and −4.97 kcal/mol, respectively. The binding energies between LYN and Anhydrobelachinal, Tetrandrine, and 11‐hydroxyrankinidine were −6.50, −5.96, and −4.69 kcal/mol, respectively. These results demonstrated that CFTR and LYN could bind spontaneously to their respective active compounds from BJJP, indicating that the anti‐fibrotic effect of BJJP may involve key compounds (e.g., kaempferol, baicalein, luteolin) targeting CFTR, as well as anhydrobelachinal, tetrandrine, and 11‐hydroxyrankinidine targeting LYN.

### 3.7 Analysis of Immune Infiltration in HF

Based on the above enrichment results of key gene pathways, we speculated that the mechanism by which BJJP treats HF might be related to the regulation of immunity by CFTR and LYN. We further employed the ssGSEA algorithm to detect immune infiltration in HF. As shown in the heat map of immune infiltration distribution in Figure S2A, there were significant differences in the composition of immune cells between the HF group and the control group. The proportion of specific immune cells (such as T cell subsets and macrophages) varied significantly between the two groups, suggesting that the occurrence and development of HF was accompanied by immune microenvironment remodeling. Figure S2B shows the heat map of immune cell differences. Compared with the control group, the abundances of cells such as T cells CD4 memory resting and macrophages M2 in the HF group were significantly altered, further clarifying the abnormal activation of immune cell subsets in the HF state and reflecting specific regulation of the immune response. The box plot of the proportion of immune cells in Figure S2C shows that the proportions of γδ T cells, macrophages M1, neutrophils, T cells CD4 memory resting, macrophages M2 and T cells follicular helper exhibited trend changes between the HF group and control group. However, there were no statistically significant differences in the proportions of mast cells activated, T cells CD8, dendritic cells resting, plasma cells, B cells naive and NK cells between the two groups.

### 3.8 Correlation Analysis of LYN, CFTR and Immune Cells

**FIGURE 6:**
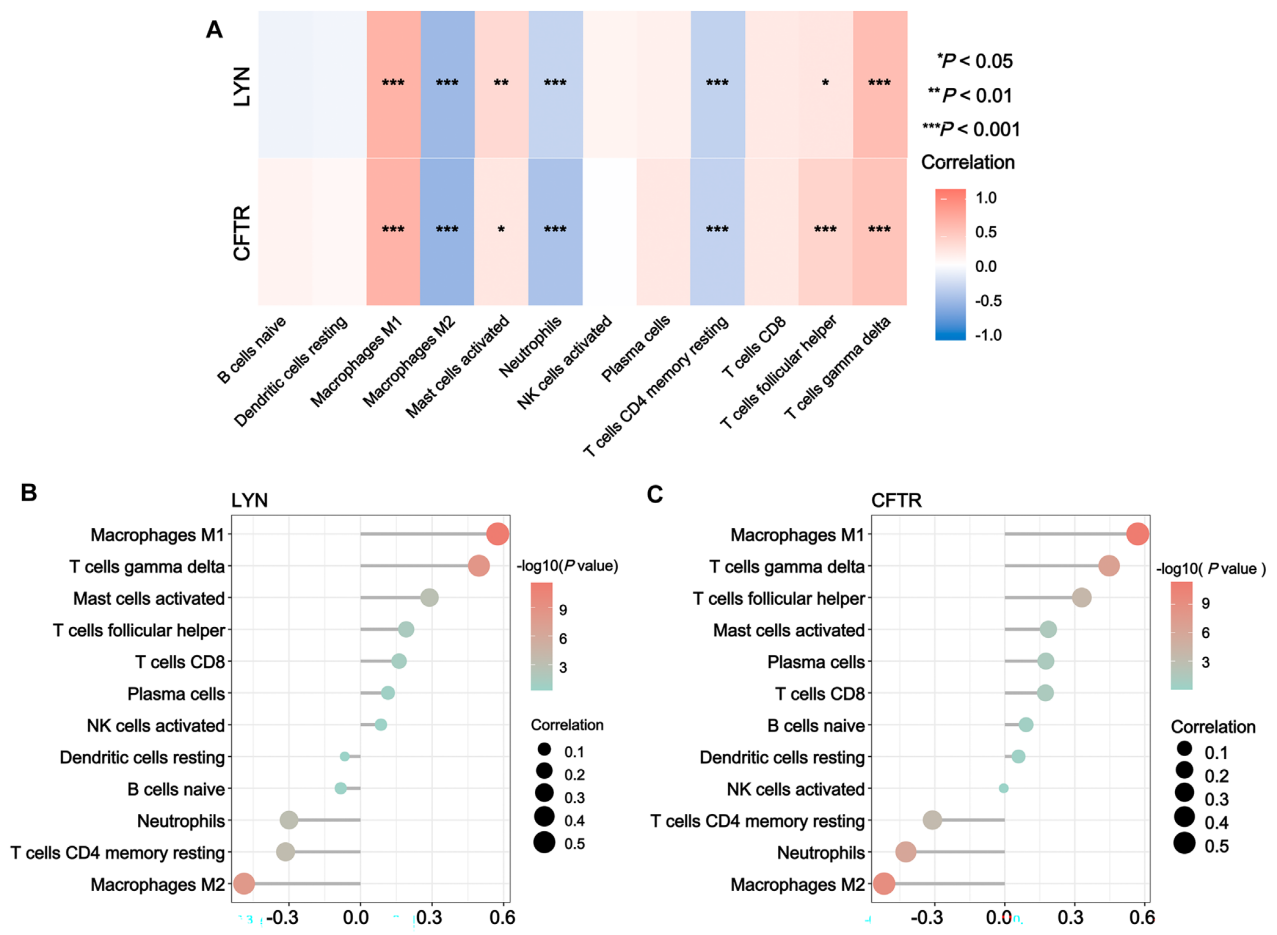
Correlation analysis of LYN, CFTR, and immune cells. (A) Correlation heatmap of LYN and CFTR genes with immune cell subsets. (B) Bubble plot of the correlation and statistical significance of LYN gene associated with immune cell subsets. (C) Bubble plot of the correlation and statistical significance of CFTR gene associated with immune cell subsets. *p < 0.05, **p < 0.01, ***p < 0.001.

As indicated by the heat map in Figure 6, which illustrates the correlations between LYN, CFTR, and immune cell subsets, both LYN and CFTR exhibited positive correlations with immune cell subsets including Macrophages M1, activated Mast cells, follicular helper T cells, and gamma delta T cells. In contrast, they showed negative correlations with Macrophages M2, Neutrophils, and CD4 memory resting T cells. These findings suggest that LYN and CFTR may participate in the remodeling of the immune microenvironment in HF by regulating the distribution of immune cell subsets.

### 3.9 Identification of Chemical Components in BJJP by UHPLC‐HRMS

To characterize the chemical components of BJJP, we analyzed the extract using a UHPLC‐HRMS platform combined with database matching (mzCloud and mzVault). After strict filtering (mass deviation < 5 ppm, matching score > 85), a total of 133 active compounds were identified (detailed information is provided in Table S2). Representative chromatograms of BJJP, including the positive‐mode total ion current chromatogram, negative‐mode total ion current chromatogram, and 254‐nm wavelength chromatogram, are presented in Figure S3.

### 3.10 BJJP Attenuated CCl_4_ ‐Induced HF In Vivo

**FIGURE 7:**
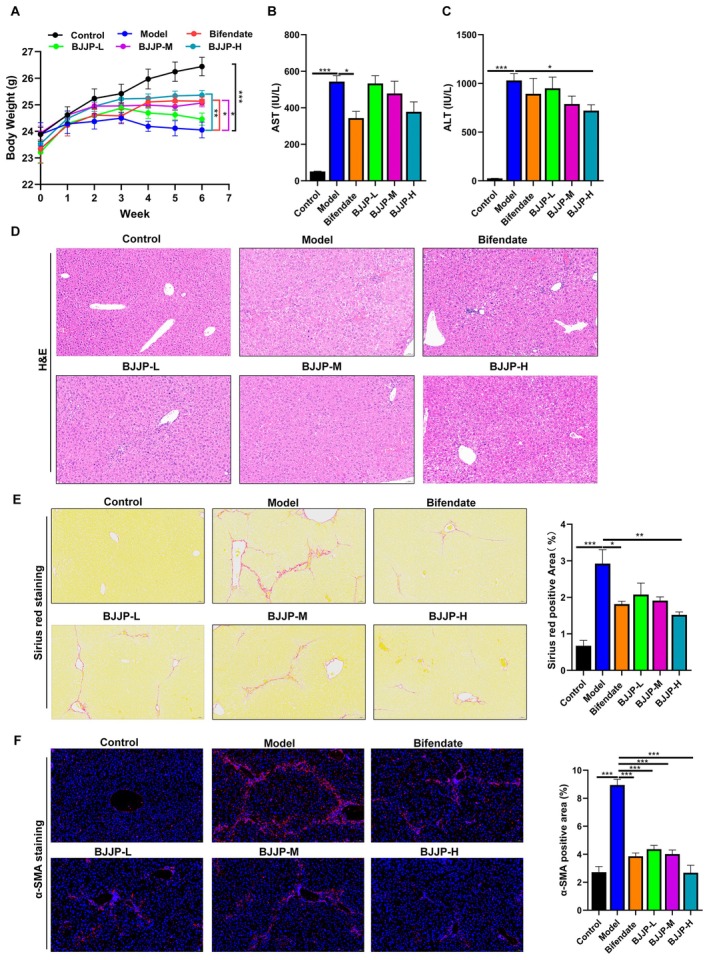
BJJP attenuated CCl_4_‐induced HF in vivo. (A) Body weight changes of mice in each group. (B) Serum AST and ALT (C) analysis. (D) The liver tissues were stained with H&E, Sirius red (E) and immunofluorescence (F) to detect α‐SMA content. *p < 0.05, **p < 0.01, ***p < 0.001. ALT, alanine aminotransferase; AST, aspartate aminotransferase; α‐SMA, α‐smooth muscle actin.

To validate BJJP's therapeutic efficacy in HF, we utilized a CCl_4_‐induced mouse model. Body weights of the mice were tracked longitudinally across model establishment, drug treatment, and various dosage interventions. As shown in Figure 7A, body weight in the control group exhibited an overall upward trend over time, while that of the HF‐model group increased relatively slowly and even tended to decrease in the later stage. The changes in body weight of the BJJP and bifendate treatment groups were between those of the control group and the model group. At the 6th week, the body weight of mice in the HF‐model group was significantly lower than that in the control group, while the body weights of mice in the BJJP‐M, BJJP‐H, and bifendate treatment groups were significantly higher than those in the HF‐model group. Further detection of serum AST and ALT levels in mice of different groups showed that both AST and ALT levels in the HF‐model group were significantly higher than those in the control group. After BJJP treatment, the serum AST and ALT levels in mice exhibited a decreasing trend (Figure 7B,C). The pathological staining results of mouse liver tissues revealed that the HF‐model group had obvious hepatic necrosis, with significant inflammatory cell infiltration, fatty vacuoles, and disrupted liver tissue structure, disordered arrangement of hepatic cords, and formation of fibrous septa in the portal area and between lobules of the liver tissue, which divided the liver tissue into “pseudolobules” (Figure 7D). The results of Sirius red‐picric acid staining also showed that in the control group, only a very small amount of red fibrous hyperplasia was observed in the portal area, the structure of hepatic lobules was clear, and no pseudolobules were formed. In the model group, a large number of red collagen fibers were found in the portal area and between lobules, forming fibrous septa, and pseudolobular structures were visible. The area of Sirius red‐positive staining was significantly larger than that in the control group (Figure 7E), which was consistent with the pathological characteristics of HF. In contrast, BJJP treatment alleviated these pathological alterations (Figure 7D,E). α‐smooth muscle actin (α‐SMA) is an important marker for HSC activation, and its expression level is closely related to the severity of HF. To further study the effect of BJJP on HF, immunofluorescence staining was used to detect the expression of α‐SMA in liver tissues. Compared with the HF‐model group, α‐SMA expression was significantly decreased in the BJJP‐treated groups (Figure 7F). Overall, BJJP treatment improved body weight and liver function in mice, and BJJP significantly attenuated CCl_4_‐induced HF.

### 3.11 Verification of Potential Target Genes of BJJP in Anti‐HF

**FIGURE 8:**
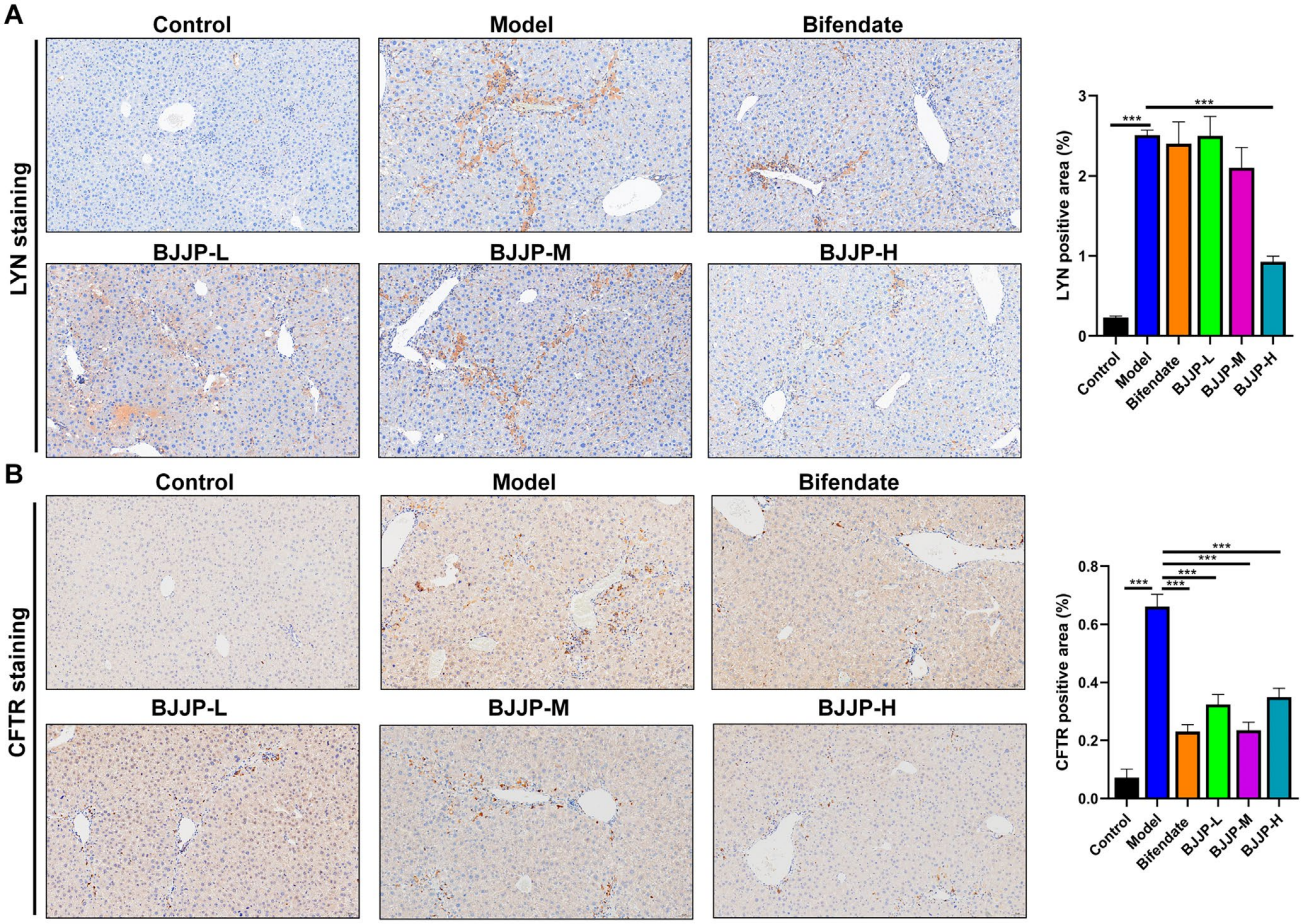
Verification of potential target genes of BJJP in anti‐HF. (A) Immunohistochemical analysis of LYN expression in liver tissues and the quantitative analysis. (B) Immunohistochemical analysis of CFTR expression in liver tissues and the quantitative analysis. ***p < 0.001.

Based on the aforementioned network pharmacology and machine learning analyses, LYN and CFTR were identified as the core therapeutic targets of BJJP for HF. Immunohistochemical staining results revealed that protein levels of LYN and CFTR in liver tissues were significantly elevated in the HF‐model group compared with the control group. Following BJJP treatment, the protein expression of both LYN and CFTR was markedly reduced. These findings suggest that BJJP may exert its anti‐HF effect by inhibiting the protein expression of LYN and CFTR (Figure 8A,B).

**FIGURE 9:**
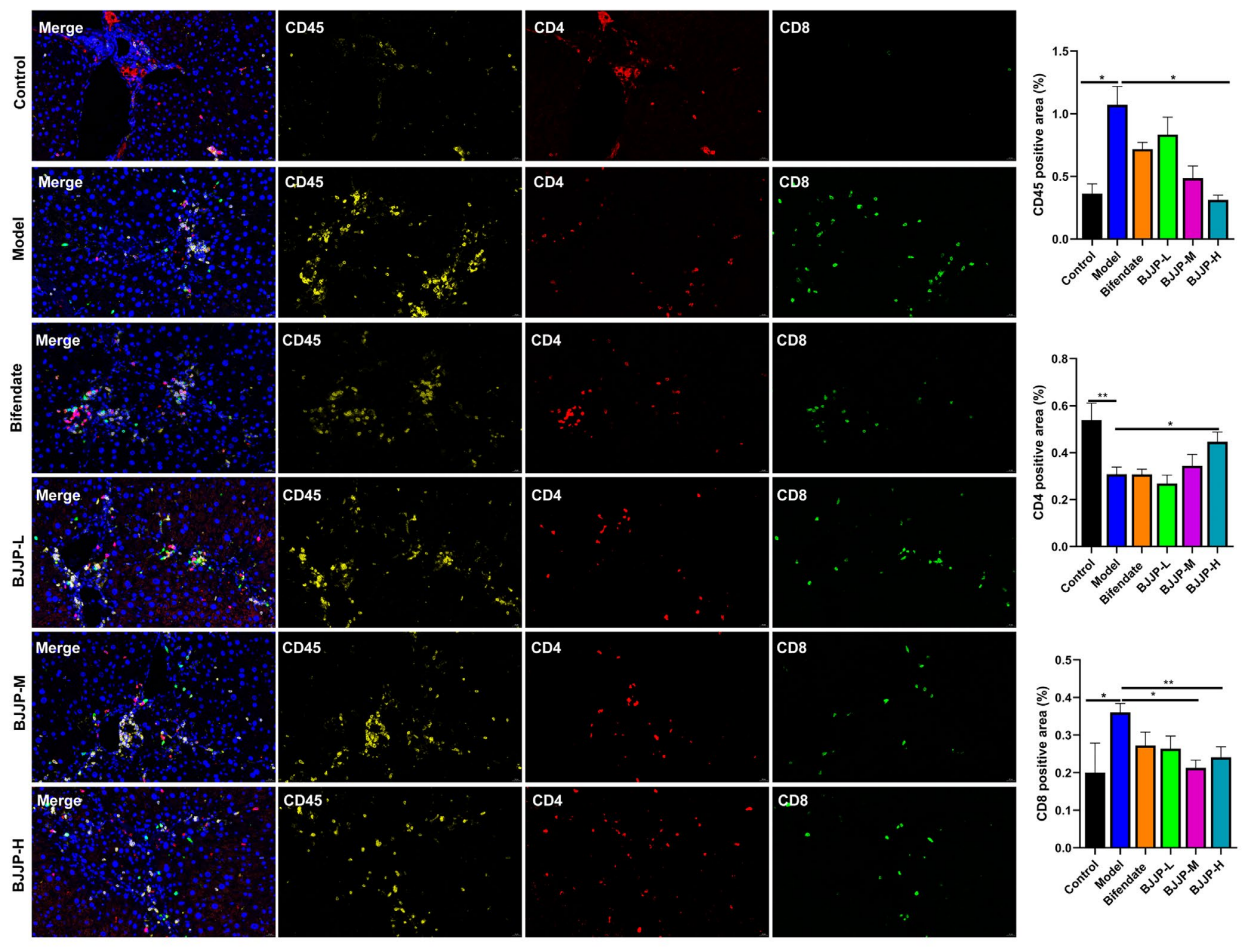
Multiplex immunohistochemical staining of the liver tissues costained with CD45 (yellow), CD4 (red), and CD8 (green). All images of immunostaining were analyzed with ImageJ software. *p < 0.05, **p < 0.01.

Multiplex immunohistochemical staining for T cell markers revealed that, relative to the control group, the HF‐model group exhibited significantly increased positive staining areas for CD45 and CD8, along with a marked reduction in the positive staining area for CD4 in mouse liver tissues. In the BJJP‐H treatment group, compared with the HF‐model group, the positive staining areas for CD45 and CD8 in liver tissues were significantly decreased, while the positive staining area for CD4 was significantly increased (Figure 9).

**FIGURE 10:**
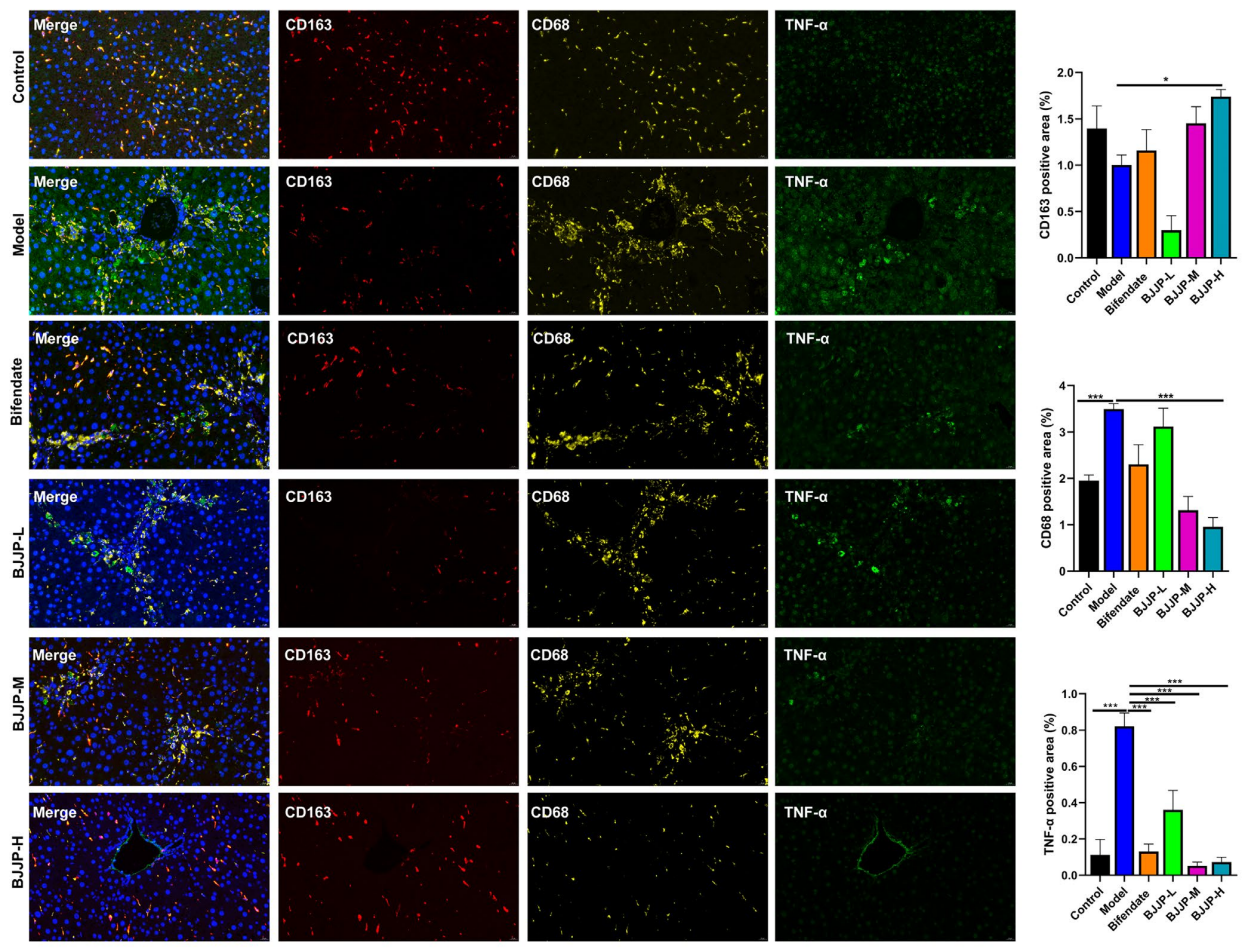
Multiplex immunohistochemical staining of the liver tissues costained with CD163 (red), CD68 (yellow), and TNF‐α (green). All images of immunostaining were analyzed with ImageJ software. *p < 0.05, ***p < 0.001.

Results of multiplex immunohistochemical staining for macrophage markers demonstrated that, in contrast to the control group, the HF‐model group showed increased positive staining areas for CD68 and TNF‐α, coupled with a decreased positive staining area for CD163 in mouse liver tissues. Following treatment with BJJP‐H, the positive staining areas for CD68 and TNF‐α in the liver tissues of HF mice were significantly reduced, whereas the positive staining area for CD163 was significantly increased (Figure 10).

## 4 Discussion

HF is a dynamic wound‐healing response to chronic insults like viral hepatitis or alcohol. Pathologically, it involves the activation of HSCs and aberrant accumulation of collagen‐rich ECM. HF serves as a pivotal, reversible checkpoint: timely intervention to remove the insult can halt fibrosis and avert the development of cirrhosis (Chen, Wu, et al. 2025; Mayorca‐Guiliani et al. 2025). However, if left unchecked, persistent injury disrupts hepatic lobular architecture, leading to pseudolobule formation and irreversible cirrhosis, which may further progress to HCC. This critical position as a “reversible–irreversible pivot” renders HF a core target for the management of chronic liver diseases, underscoring the urgency of early diagnosis and intervention. Nevertheless, effective clinical strategies specifically targeting HF remain limited (Lin et al. 2025).

Studies in recent years have shown that TCM prescriptions have potential in the prevention and treatment of HF (Liang, Liu, et al. 2025). BJJP is a classic formula in TCM renowned for replenishing qi to strengthen healthy qi, activating blood circulation to resolve stasis, and softening hard masses to dissipate nodules. Clinically, it is widely prescribed for liver diseases, including HF and HCC (Chen, Wu, et al. 2025; Chen, He, et al. 2025; Cheng et al. 2023). However, due to the diversity of TCM‐targets, the molecular mechanism of BJJP in the treatment of HF remains to be further explored. Therefore, this study integrated bioinformatics approaches (network pharmacology, machine learning, and molecular docking) to systematically explore the active compounds, potential therapeutic effects, and key targets of BJJP, which were further verified through in vivo experiments. We identified CFTR and LYN as core therapeutic targets of BJJP against HF, systematically analyzing their expression, prognostic value, and correlation with immune infiltration. Molecular docking with six key active compounds of BJJP demonstrated strong binding affinities. These results suggest that BJJP exerts its anti‐fibrotic effects by downregulating CFTR and LYN expression through constituent compounds (e.g., kaempferol, baicalein, and luteolin). This mechanism, in turn, modulates intrahepatic immune microenvironment remodeling, inhibits HSCs activation, and retards the progression of HF.

Cystic fibrosis transmembrane conductance regulator (CFTR) is a member of the ATP‐binding cassette (ABC) transporter family. Its main function is to act as a cAMP‐dependent chloride ion channel, regulating ion balance inside and outside cells (Eldredge et al. 2024). Studies have shown that CFTR is expressed on the luminal membrane of cholangiocytes, regulating the alkalinity and fluidity of bile. Dysfunction of CFTR can lead to reduced bile secretion and accumulation of bile acids, which in turn cause toxic damage and chronic inflammation of cholangiocytes, ultimately resulting in HF (Fiorotto and Strazzabosco 2019). In addition to its role as an anion channel, CFTR is also a key coordinator of protein network homeostasis. In the dynamic systems of interacting proteins, CFTR establishes connections and is influenced by the protein homeostasis network (Carlile et al. 2022; Villella et al. 2013). Zhang et al. found that CFTR was highly expressed in the liver tissues of both patients with HF and fibrotic model mice. It was also confirmed that CFTR could activate autophagy by targeting G3BP1, promote the activation of HSCs and the progression of HF, and was involved in the TGF‐β/Smad2/3 signaling pathway. Therefore, CFTR was considered a potential therapeutic target for HF (Zhang et al. 2025). NASH is regarded as a critical stage in the progression of NAFLD and increases the risk of end‐stage liver diseases such as HF, cirrhosis, and HCC. Rajak S et al. (Rajak et al. 2023) uncovered CFTR as a DEG in the livers of human NASH patients, and upregulated hepatic CFTR expression was similarly observed in two mouse models of diet‐induced NASH. This study demonstrated that CFTR was highly expressed in the liver tissues of mice with CCl_4_‐induced HF. Following treatment with BJJP, both liver function and the severity of HF in these mice were significantly ameliorated, which was accompanied by reduced hepatic CFTR expression. These findings suggest that BJJP may alleviate HF by inhibiting CFTR expression.

LYN kinase, a non‐receptor tyrosine kinase belonging to the Src kinase family, is part of the broader category of tyrosine kinases, which encompass both receptor and non‐receptor types (de Jesus et al. 2023). Previous studies have demonstrated that tyrosine kinases are linked to HF through their regulation of HSCs. Specifically, during the progression of HF, the expression levels of several receptor tyrosine kinases, including platelet‐derived growth factor receptor (PDGFR), vascular endothelial growth factor receptor (VEGFR), and fibroblast growth factor receptor (FGFR), are significantly elevated, while activated hematopoietic stem cells have been associated with increased expression of numerous tyrosine kinases (Qu et al. 2015, 2016). LYN has also been reported to be highly expressed in liver tissues of mice with CCl_4_‐induced HF, and its overexpression markedly promotes the expression of HSC activation markers both in vivo and in vitro (Li et al. 2017). Pham H et al. identified hyperactive LYN signaling in myofibroblasts derived from patients with fibrotic chronic pancreatitis. LYN activation colocalizes with activated myofibroblast markers and exhibits an approximately 11‐fold upregulation in chronic pancreatitis tissues compared with normal counterparts. In a murine model of chronic pancreatitis‐induced fibrosis, LYN inhibition abrogates procollagen and collagen synthesis in myofibroblasts. They propose that LYN, functioning as a positive regulator of myofibroblast migration, proliferation, and collagenogenesis, represents a key therapeutic target for fibrosis prevention (Pham et al. 2016). Our findings demonstrated that LYN exhibited favorable diagnostic efficacy in predicting HF and was significantly upregulated in liver tissue samples from HF mice. Additionally, treatment with BJJP reduced LYN expression levels in the liver tissues of HF mice. To further elucidate the regulatory effects of BJJP on CFTR and LYN, molecular docking was performed between the major components of BJJP and these two proteins. Results demonstrated that key compounds, kaempferol, baicalein, and luteolin, exhibited strong binding affinity for CFTR, with luteolin displaying the highest affinity. For LYN, major compounds including anhydrobelachinal, tetrandrine, and 11‐hydroxyrankinidine also demonstrated robust binding affinity, among which anhydrobelachinal showed the strongest interaction. These findings further validate that BJJP exerts regulatory effects on CFTR and LYN through its active compounds.

Long‐term chronic low‐grade inflammation and the activation of the innate and adaptive immune systems play crucial roles in all aspects of HF pathogenesis (Fan et al. 2025; Gao et al. 2025). Using ssGSEA analysis, we discovered that the proportion of specific immune cells (such as T cell subsets and macrophages) varied significantly between the two groups, suggesting that the development of HF was accompanied by the remodeling of the immune microenvironment. CD45, a protein tyrosine phosphatase expressed on the surface of almost all hematopoietic cells except red blood cells, primarily functions as a marker of immune cell infiltration, and changes in its expression reflect the activity of liver inflammation and immune responses. In diverse HF models, including bile duct ligation (BDL) or CCl_4_‐induced liver injury models, CD45‐positive cells are significantly increased in liver tissue, particularly in the periportal region (R. Zhang, Kikuchi, et al. 2019). Multiple studies have elucidated the dynamic changes and functional discrepancies of CD4^+^ and CD8^+^T cell subsets during the progression and prognosis of HF (Zheng et al. 2025). In liver tissue, the infiltration of cytotoxic CD8^+^T cells is elevated, and dysfunction of CD4^+^T cells can induce fibrosis (Liang et al. 2020). In the CCl_4_ model, the frequency of hepatic CD8^+^T cells was 2.6‐fold higher than that in the control group, indicating marked infiltration (Zhang et al. 2017). It was reported that liver fibrosis was accompanied by an immune tolerance state, where the CD4^+^/CD8^+^ ratio decreased as the proportion of CD4^+^ cells decreased and that of CD8^+^ cells increased (Albillos et al. 2022; Guo et al. 2024; Zhao et al. 2022). It has been reported that from the early to the middle stage of fibrosis, the number of CD68^+^ macrophages increases significantly, to approximately 2–3 times that in normal liver, and these cells are mainly concentrated in the portal area, fibrous septa, and scar regions. CD68^+^ macrophages secrete inflammatory factors such as TNF‐α, activate HSCs, and promote collagen deposition. In the portal hypertension model, CD68^+^macrophages colocalized with fibroblasts, express Spp1 (osteopontin), and directly promote fibrosis in the portal area (Lebedeva 2023; Liu et al. 2010; Ma et al. 2025). CD163 is a surface molecule expressed on monocytes/macrophages and functions as a high‐affinity scavenger receptor for the hemoglobin‐haptoglobin complex. Upon activation, monocytes/macrophages release CD163 into the circulation in the form of soluble CD163 (sCD163). As a marker of monocyte/macrophage activation, sCD163 exhibits a negative correlation with the expression level of membrane‐bound CD163 (Davis and Zarev 2005; Skytthe et al. 2023). A growing body of research strongly suggests that CD163 plays a direct role in anti‐inflammatory processes, as its expression and function are regulated by multiple mediators (Droste et al. 1999; Hintz et al. 2002). Xie P et al. demonstrated that CD163, which is associated with monocytes/macrophages, serves as an indicator of liver inflammation and the severity of liver cirrhosis. Specifically, in classical monocytes, the mean fluorescence intensity of CD163 expression was significantly higher in healthy individuals than in patients with chronic hepatitis B (Xie, Xie, et al. 2022; Xie, Yao, et al. 2022). Liver biopsy results revealed that CD163 levels in liver tissues were significantly lower in patients with advanced HF than in those with mild HF (Saldarriaga et al. 2024). Animal experiments have also demonstrated that CD163 is significantly downregulated in liver tissues of mice with CCl_4_‐induced HF. Furthermore, drug treatment can alleviate HF by upregulating CD163 expression in the liver tissues of these fibrotic mice (Jia et al. 2025; Qi et al. 2023). Our research findings demonstrate that BJJP exerts an effective regulatory effect on the distribution of T cells and macrophages in the context of HF. In our in vivo studies, we observed that in the fibrotic liver microenvironment, the T cell markers CD45 and CD8, along with the macrophage markers CD68 and TNF‐α, were significantly upregulated, whereas the proportions of CD4^+^ T cells and CD163^+^ macrophages were markedly decreased. Notably, BJJP administration reversed the expression levels of these immune cell markers in liver fibrotic tissues. Collectively, these data highlight the therapeutic potential of BJJP in shifting the intrahepatic immune microenvironment of HF from a profibrotic state toward a reparative phenotype, thereby offering a novel strategy for the management of HF.

## 5 Conclusion

This study systematically explored the anti‐fibrotic mechanisms of BJJP in treating HF using an integrated approach combining network pharmacology, machine learning, and experimental validation. We identified two signature genes (LYN and CFTR) as key targets of BJJP, which are closely associated with immune microenvironment remodeling in HF. UHPLC‐HRMS characterized 133 active compounds in BJJP, and molecular docking confirmed strong binding affinities between core compounds (e.g., kaempferol, baicalein, luteolin) and LYN/CFTR. In vivo experiments verified that BJJP effectively ameliorates CCl_4_‐induced HF by downregulating LYN/CFTR expression, restoring T cell subset homeostasis (reducing CD45^+^/CD8^+^ T cell infiltration and increasing the proportion of CD4^+^ T cells), and modulating macrophage polarization (shifting from profibrotic M1 toward anti‐fibrotic M2 macrophages). Collectively, these results demonstrate that BJJP exerts therapeutic effects on HF via the “active compound‐target‐immune regulation” axis. This study provides a scientific basis for the clinical application of BJJP and a novel strategy for exploring the mechanisms of TCM formulas. Limitations include the focus on a single HF animal model; future studies should validate the findings in multiple models and clinical cohorts to improve generalizability.

## Author Contributions

**Yuhong Song:** supervision, data curation, writing – review and editing. **Chan Mo:** conceptualization, writing – review and editing, writing – original draft, funding acquisition. **Yuan Liu:** software, project administration. **Jiaorong Zheng:** investigation. **Min Hong:** writing – review and editing, resources. **Zhuolin Wei:** methodology. **Jinnan He:** visualization.

## Funding

This work was supported by the National Natural Science Foundation of China (82204841), the Natural Science Foundation of Guangdong Province (2025A1515010076, 2021A1515110861), and the Shenzhen Medical Research Fund (A2303020).

## Ethics Statement

All animal experiments were conducted in accordance with the protocols approved by the Laboratory Animal Ethics Committee of Shenzhen Zhongxun Precision Medicine Research Institute and compliance with the relevant ethical regulations for animal research (Approval number: ZXJZ202401010008).

## Conflicts of Interest

The authors declare no conflicts of interest.

## Supporting information


**Table S1:** Medicines included in the BJJP.
**Table S2:** The characteristic components of Biejia Decoction Pills were identified and analyzed based on LC–MS.
**Figure S1:** Constructing the regulatory network of BJJP against HF.
**Figure S2:** Analysis of immune infiltration in HF by ssGSEA.
**Figure S3:** The identification result of the chemical compounds of BJJP by UHPLC‐HRMS.
**Method S1:** Acquisition of BJJP‐related active compounds and target genes.
**Method S2:** Acquisition of HF‐related targets and analysis by multiple HF‐related databases.
**Method S3:** Identification of characteristic genes of HF lesions.
**Method S4:** Molecular docking.
**Method S5:** Characterization of primary chemical constituents of BJJP by UHPLC‐HRMS.
**Method S6:** H&E and Sirius Red Staining of mouse liver tissue paraffin sections.
**Method S7:** Immunohistochemical staining for LYN and CFTR expression.
**Method S8:** Detailed procedures for immunofluorescence staining of mouse liver tissue paraffin sections.

## Data Availability

The data that support the findings of this study are available from the corresponding author upon reasonable request.
