# Supplementary material for: Unveiling the Immunopharmacological Mechanisms of Biejiajian Pills (BJJP) Underlying the Treatment of Hepatic Fibrosis: Insights From Network Pharmacology and Experimental Validation
Source: Food Sci Nutr. 2026 Mar 23;14(3):e71683. doi: 10.1002/fsn3.71683 (PMC13093626; doi:10.1002/fsn3.71683)
Supplement: Supplementary file 1 — Table S1: Medicines included in the BJJP. Table S2: The characteristic components of Biejia Decoction Pills were identified and analyzed based on LC–MS. Figure S1: Constructing the regulatory network of BJJP against HF. Figure S2: Analysis of immune infiltration in HF by ssGSEA. Figure S3: The identification result of the chemical compounds of BJJP by UHPLC‐HRMS. Method S1: Acquisition of BJJP‐related active compounds and target genes. Method S2: Acquisition of HF‐related targets and analysis by multiple HF‐related databases. Method S3: Identification of characteristic genes of HF lesions. Method S4: Molecular docking. Method S5: Characterization of primary chemical constituents of BJJP by UHPLC‐HRMS. Method S6: H&E and Sirius Red Staining of mouse liver tissue paraffin sections. Method S7: Immunohistochemical staining for LYN and CFTR expression. Method S8: Detailed procedures for immunofluorescence staining of mouse liver tissue paraffin sections. [file FSN3-14-e71683-s001.docx]

**Unveiling the immunopharmacological mechanisms of Biejiajian Pills (BJJP) underlying the treatment of hepatic fibrosis: insights from network pharmacology and experimental validation**

Chan Mo^1^ Zhuolin Wei^1^ Jinnan He^1^ Yuan Liu^3^  Jiaorong Zheng^1^ Min Hong^1*^  Yuhong Song^2*^

^1^Department of First Clinical Medical College, Guangdong Pharmaceutical University, Guangzhou, Guangdong, 510006, PR China

^2^School of Traditional Chinese Medicine, Guangdong Pharmaceutical University, Guangzhou, Guangdong, 510006, PR China

^3^College of Integrated Traditional Chinese and Western Medicine, Jining Medical University, Jining, Shandong, 272067, PR China

^*^Corresponding author: Dr Min Hong, No.19, Nonglinxia Road, Yuexiu District, Guangzhou, 510080, PR China, Email address: hongmin@gdpu.edu.cn. Dr Yuhong Song, No. 280, Outer Ring East Road, University Town, Panyu District, Guangzhou, 510006, PR China, Email address: songyuhong@gdpu.edu.cn.

***Supplementary Method***

**Supplementary Method S1: Acquisition of BJJP-related active compounds and target genes.**

Using the Traditional Chinese Medicine Systems Pharmacology Database (TCMSP, https://old.tcmsp-e.com/tcmsp.php), drug compounds of BJJP were retrieved with the filtering criteria of oral bioavailability (OB) > 30% and drug-likeness (DL) > 0.18. Supplementary compounds were retrieved from the Encyclopedia of Traditional Chinese Medicine (ETCM) to improve the coverage of TCM-specific compounds. Subsequently, the corresponding Simplified Molecular-Input Line-Entry System (SMILES) notations of these compounds were obtained from the PubChem Compound Database (https://pubchem.ncbi.nlm.nih.gov/).

SwissTargetPrediction (http://www.swisstargetprediction.ch/), a small molecule structure-based target prediction tool, was used to supplement TCMSP’s target coverage. The acquired SMILES notations were imported into this platform, and target genes were filtered with the criterion of a prediction probability > 0.

All predicted targets from TCMSP and SwissTargetPrediction were aggregated into a preliminary dataset. Gene symbols were standardized to HGNC- (HUGO Gene Nomenclature Committee) approved names; duplicate targets were removed by retaining unique gene symbols, and non-human targets (e.g., mouse, rat) were excluded to ensure relevance to human hepatic fibrosis (HF).

**Supplementary Method S2: Acquisition of HF-related targets and analysis by multiple HF-related databases.**

HF-related target genes were retrieved from three complementary databases: GeneCards (https://www.genecards.org/): a comprehensive gene-disease association database, was used to acquire all known HF-related targets with the filtering criteria of a relevance score > 1 and protein-coding genes to expand the disease target pool. OMIM (Online Mendelian Inheritance in Man): a human genetic disease database, was used to mine core pathogenic genes of HF and screen disease-specific targets (no additional filtering thresholds beyond inherent curation standards). ETCM: a TCM-specialized database, was used to supplement HF-related targets and enhance consistency with TCM compound-target information (no additional filtering thresholds applied).

Targets from GeneCards, OMIM, and ETCM were compiled into a combined dataset. Gene symbols were standardized to HGNC nomenclature to ensure consistency; duplicate targets were eliminated by retaining unique gene symbols (regardless of database source), and uncharacterized transcripts or targets without functional annotations were excluded based on Ensembl and NCBI gene annotations. Differential expression analysis between the HF group and normal control group was performed using the R package “limma” (Version 3.52.4; https://bioconductor.org/packages/release/bioc/html/limma.html), which calculated the corresponding adjusted P-values (adj. P-value) and log_2_ fold change (log_2_FC) for each gene. Differentially expressed genes (DEGs) were screened based on two criteria: log_2_FC > 0.263 and adj. P-value < 0.05.

These databases were selected for their complementary strengths: TCMSP and ETCM for TCM compound screening, SwissTargetPrediction for structure-based target prediction, and GeneCards/OMIM for the comprehensive and specific mining of HF-related genes, thus ensuring the comprehensive and reliable collection of targets.

**Supplementary Method S3: Identification of characteristic genes of HF lesions.**

Three machine learning algorithms, LASSO, RF, and SVM-RFE were employed to further screen potential characteristic genes for diagnosing HF lesions. LASSO, a dimensionality reduction method, exhibits advantages over regression analysis in evaluating high-dimensional data. In LASSO analysis, regularization penalty parameters were used to select feature variables via 10-fold cross-validation. Within the SVM-RFE algorithm (a supervised machine learning approach), recursive feature elimination (RFE) was applied to rank the characteristic genes associated with HF lesions; predictive performance was evaluated through 10-fold cross-validation to identify definitive characteristic genes. RF outperforms linear discriminant analysis and the mean squared error method in selecting relevant features and eliminating redundant ones. Feature selection was conducted using 10-fold cross-validation, with genes demonstrating a relative importance (MeanDecreaseGini) greater than 0.25 designated as important variables (Yu, Wang, Han, & He, 2012). LASSO regression, SVM-RFE, and RF analyses were implemented using the R packages “glmnet” (version 4.1-6, <https://cran.r-project.org/web/packages/glmnet/index.html)> (Mceligot, Poynor, Sharma, & Panangadan, 2020), “e1071” (version 1.7-14, <https://cran.r-project.org/web/packages/e1071/index.html)> (Sanz, Valim, Vegas, Oller, & Reverter, 2018), and “randomForest” (version 4.7-1.1, <https://cran.r-project.org/web/packages/randomForest/)> (Utkin & Konstantinov, 2022), respectively. Genes identified as the intersection of these three methods were designated as key diagnostic characteristic genes for HF lesions.

**Supplementary Method S4: Molecular Docking.**

The crystal structures of the characteristic gene-encoded proteins were retrieved from the Protein Data Bank (PDB, <https://www.rcsb.org/)> (Reimand et al., 2019). For CFTR (UniProt ID: P13569), we selected the crystal structure of the minimal human CFTR first nucleotide-binding domain (NBD1) as a head-to-tail dimer (PDB ID: 2PZE) (Atwell et al., 2010). The docking pocket was defined as the nucleotide-binding site of NBD1, with the grid center set to X=29.1 Å, Y=33.4 Å, Z=41.7 Å and a grid size of 22 Å × 22 Å × 22 Å to fully cover the functional domain. For LYN (UniProt ID: P07948), we used the crystal structure of the human Lyn SH3 domain (PDB ID: 6NMW) (Berndt, Gurevich, & Iverson, 2019). PyMOL was employed to remove water molecules and original ligands from the protein structure; the target proteins were then selected based on their corresponding mRNA-encoded sequences. The processed proteins were subsequently imported into AutoDock Tools for hydrogenation, charge calculation, and the combination of non-polar hydrogen atoms. Next, appropriate Grid Box dimensions and genetic algorithm parameters were configured in AutoDock Vina to perform molecular docking. Finally, the molecular docking results were visualized using Discovery Studio 2019 software.

**Supplementary Method S5: Characterization of primary chemical constituents of BJJP by UHPLC-HRMS.**

Two grams of BJJP (Sinopharm Group Zhonglian Pharmaceutical Co., Ltd) were weighed, crushed, and mixed with 40 mL of 80% methanol aqueous. Ultrasonic extraction was performed for 30 mins. Then, 1 mL of the BJJP test sample was accurately measured, thoroughly mixed, and filtered through a 0.22 μm filter membrane. The resulting filtrate was used as the test solution.The instrument parameters and detection conditions were as follows, chromatographic column: Xtimate UHPLC C18 (2.1×100 mm, 1.8 μm), the mobile phase A was set as 0.1% formic acid-acetonitrile, and phase B as 0.1% formic acid-water with a flow rate of 0.3 mL/min. Full-wavelength scanning was performed with the column temperature maintained at 35 ℃, an injection volume of 2 μL, an ESI ion source, a spray voltage of ±3.5 kV/-2.8 kV, a sheath gas temperature of 350℃ and flow rate of 50 Arb, an auxiliary gas flow rate of 8 Arb, a purge gas flow rate of 1 Arb, a nebulizer temperature of 350℃, an ion transfer tube temperature of 325℃. Positive and negative ion modes were scanned separately over the m/z range of 100 to 1500 and the collision energy gradient set at 20%, 40%, and 60%. The collected raw data were imported into Compound Discoverer 3.3 software, where the identification process for unknown compounds was established using its wizard settings and method templates, followed by peak extraction from the raw data. Characteristic peaks in the samples were analyzed, and candidate molecular formulas were generated from the extracted molecular ion chromatographic peaks and isotope peaks, which were then matched against databases including mzCloud and mzVault. After filtering results with a mass deviation exceeding 5 ppm and a matching score above 85 points, comprehensive analysis and identification were conducted.

**Supplementary Method S6: H&E and Sirius Red Staining of Mouse Liver Tissue Paraffin Sections.**

The liver tissues of mice were fixed with 4% paraformaldehyde, followed by gradient dehydration and embedding before being sectioned at a thickness of approximately 4 μm. The sections were conventionally dewaxed to water. They were stained with hematoxylin solution for several minutes, with the floating color washed off using tap water. After differentiation with hydrochloric acid-ethanol, the sections were blued. Subsequently, they were stained with eosin solution, dehydrated through gradient ethanol, cleared with xylene, and mounted with neutral gum to complete H&E staining. For Sirius Red staining, after dewaxing to water, the sections were stained in Sirius Red solution for 8 mins, then dehydrated via gradient ethanol, cleared with xylene, and mounted with neutral gum to finish the procedure.

**Supplementary Method S7: Immunohistochemical Staining for LYN and CFTR Expression.**

The liver tissue sections of mice were first dewaxed and rehydrated using dewaxing solution and anhydrous ethanol. Antigen retrieval was performed with citric acid antigen retrieval solution (pH 6.0), and after natural cooling, the sections were washed with PBS (pH 7.4). They were then incubated in 3% hydrogen peroxide solution at room temperature in the dark, followed by PBS washing to block endogenous peroxidase. Then, 3% BSA was added to the histochemical circle for blocking at room temperature. The blocking solution was removed, and primary antibodies, including LYN (clone 3C7F2, 1:1000, mouse, Proteintech) and CFTR (1:2000, rabbit, Servicebio) were added, with incubation carried out overnight in a wet box at 4°C. After PBS washing, the corresponding HRP-labeled secondary antibodies were added and incubated at room temperature. The sections were washed again with PBS, fresh DAB chromogenic solution was added, and the reaction was monitored under a microscope before being rinsed with tap water to stop color development. Subsequent steps included hematoxylin counterstaining, differentiation, bluing, and rinsing with running tap water. After a series of dehydration and clearing treatments with gradient ethanol, n-butanol, and xylene, the sections were slightly dried and sealed with mounting medium. Finally, they were examined under a white light microscope for result interpretation. Nuclei stained with hematoxylin appeared blue, while positive expressions indicated by DAB were brownish-yellow.

**Supplementary Method S8: Detailed Procedures for Immunofluorescence Staining of Mouse Liver Tissue Paraffin Sections.**

For α‑SMA immunofluorescence staining of mouse liver tissue paraffin sections, the procedures were as follows: The sections were routinely dewaxed to water. Antigen retrieval was carried out with citric acid antigen retrieval solution, and after natural cooling, they were washed with PBS. After being spun dry, blocking solution was added for blocking for 30 minutes. Then the prepared primary antibody (Anti-α-SMA, 1:500, rabbit, Servicebio) was added, and the sections were laid flat in a wet box and incubated overnight at 4 °C. The next day, they were washed with PBS, and the corresponding secondary antibody was added, followed by incubation at room temperature in the dark for 30 minutes. They were washed again with PBS, and DAPI staining solution was added for incubation at room temperature in the dark for 10 mins to counterstain the cell nuclei. Finally, the slides were mounted with anti‑fluorescence quenching mounting medium, and images were collected using 3DHISTECH (Pannoramic MIDI) scanner.

The fluorescence four-marker staining steps for paraffin sections of mouse liver tissue were as follows: Paraffin sections were routinely dewaxed to water, and antigen retrieval was performed with citric acid antigen retrieval solution (pH 6.0). After natural cooling, they were washed with PBS (pH 7.4). The sections were slightly spun dry, circled with an immunohistochemical pen, and incubated with 3% hydrogen peroxide solution in the dark to block endogenous peroxidase, followed by washing with PBS. Then, the first serum blocking was conducted (10% rabbit serum or 3% BSA was selected based on the source of the primary antibody). Subsequently, four groups of processes were carried out in sequence: adding the primary antibody (incubated overnight at 4 ℃) → adding the corresponding HRP-labeled secondary antibody (incubated at room temperature for 50 mins) → adding the corresponding TSA (incubated at room temperature in the dark for 10 mins, then washed with TBST) → antibody elution (incubated at 37 ℃ for 30 mins, then washed with TBST) → serum blocking. After the four-marker primary antibody staining was completed, the cell nuclei were counterstained with DAPI (incubated at room temperature in the dark for 10 mins, then washed with PBS), and autofluorescence quenching was performed (quenching agent was added for 5 mins, followed by rinsing with running water for 10 mins). Finally, the sections were washed with PBS, sealed with anti‑fluorescence quenching mounting medium, and images were acquired based on the fluorescein parameters. A panoramic scan was performed using 3DHISTECH (Pannoramic MIDI) scanner. The following primary antibodies were used: anti-CD45(1:300, rabbit, Servicebio), anti-CD4 (1:250, rabbit, Servicebio), anti-CD8 (1:400, rabbit, Servicebio), anti-CD163 (1:500, rabbit, Servicebio), anti-CD68(1:250, rabbit, Servicebio) and anti-TNF-α (1:250, rabbit, Servicebio).

***Supplementary Tables***

**Supplementary Table S1. Medicines included in the BJJP.**

| Academic name* | English name | Chinese name | Crude material weight(g)# | Proportion(%) |
| --- | --- | --- | --- | --- |
| *Trionyx sinensis Wiegmann* | Trionycis Carapax | Biejia | 18 | 2.22 |
| *Equus asinm L.* | Asini Crorii Colla | Ejiao | 30 | 3.71 |
| *Parapolybia varia Fabricius* | Vespae Nidus | Fengfang | 40 | 4.95 |
| *Armadillidium vulgare Latreille* | Pillbug | Shufuchong | 30 | 3.71 |
| *Eupolyphaga sinenis Walker* | Eupolyphaga Steleophaga | Tubiechong | 50 | 6.19 |
| *Catharsius molossus Linnaeus* | Dung Beetle | Qianglang | 60 | 7.43 |
| *Potassium nitrate* | Niter | Xiaoshi | 120 | 14.85 |
| *Bupleurum chinense DC.* | Bupleuri Radix | Chaihu | 60 | 7.43 |
| *Scutellaria baicalensis Georgi* | Scutellariae Radix | Huangqin | 30 | 3.71 |
| *Pinellia ternate(Thunb.) Makino* | Pinelliae Rhizoma | Banxia | 10 | 1.24 |
| *Codonopsis pilosula (Franch.) Nannf* | Codonopsis Radix | Dangshen | 10 | 1.24 |
| *Zingiber officinale Roscoe* | Zingiberis Rhizoma | Ganjiang | 30 | 3.71 |
| *Magnolia officinalis Rehder & E.H.Wilson* | Magnoliae Officinalis Cortex | Houpo | 30 | 3.71 |
| *Cinnamomum cerum J.Presl* | Cinnamomi Ramulus | Guizhi | 30 | 3.71 |
| *Paeonia lactiftora Pall.* | Paeoniae Radix Alba | Baishao | 50 | 6.19 |
| *Belamcanda chinensis (L.) DC* | Belamcandae Rhizoma | Shegan | 30 | 3.71 |
| *Prunus persica (L.) Batsch* | Persicae Semen | Taoren | 20 | 2.48 |
| *Paeonia suffruticosa Andrews* | Moutan Cortex | Mudanpi | 50 | 6.19 |
| *Rheum officinale Baill* | Rhei Radix et Rhizoma | Dahuang | 30 | 3.71 |
| *Campsis grandiflora (Thunb.) K. Schum* | Campsis Flos | Linxiaohua | 20 | 2.48 |
| *Lepidtum apetalum Willd* | Descurainiae Semen | Tinglizi | 10 | 1.24 |
| *Pyrrosia sheareri (Baker) Ching* | Pyrrosiae Folium | Shiwei | 30 | 1.24 |
| *Dianthus superbus L* | Dianthi Herba | Qumai | 20 | 2.48 |

**Supplementary Table S2. The characteristic components of Biejia Decoction Pills were identified and analyzed based on LC-MS.**

| No. | Name | Chemical formula | ppm | Calc. MW | RT [min] | m/z |
| --- | --- | --- | --- | --- | --- | --- |
| 1 | α,α-Trehalose | C_12_H_22_O_11_ | -1.17 | 342.11581 | 0.827 | 341.10846 |
| 2 | Arabic acid | C_5_ H_10_O_6_ | -1.26 | 166.04753 | 0.866 | 165.04025 |
| 3 | Xylitol | C_5_H_12_O_5_ | -1.03 | 152.06832 | 0.874 | 151.06105 |
| 4 | Uric acid | C_5_H_4_ N_4_O_3_ | -1.04 | 168.02817 | 0.922 | 167.02089 |
| 5 | 4-Oxoproline | C_5_H_7_NO_3_ | -0.53 | 129.04252 | 0.971 | 128.03525 |
| 6 | L-(-)-Malic acid | C_4_ H_6_ O_5_ | -0.61 | 134.02144 | 1.112 | 133.01416 |
| 7 | Malonic acid | C_3_H_4_O_4_ | -0.56 | 104.0109 | 1.152 | 103.00362 |
| 8 | trans-Aconitic acid | C_6_H_6_O_6_ | -1.17 | 174.01623 | 1.163 | 173.00896 |
| 9 | Succinic acid | C_4_H_6_O_4_ | -0.9 | 118.0265 | 1.296 | 117.01923 |
| 10 | Gallic acid | C_7_ H_6_ O_5_ | -1.23 | 170.02131 | 1.59 | 169.01404 |
| 11 | N-Isovalerylglycine | C_7_H_13_NO_3_ | -0.71 | 159.08943 | 2.053 | 158.08215 |
| 12 | Vanillic acid 4-β-D-glucoside | C_14_H_18_O_9_ | -0.57 | 330.09489 | 2.443 | 329.08762 |
| 13 | Gentisic acid | C_7_H_6_O_4_ | -0.79 | 154.02649 | 2.954 | 153.01921 |
| 14 | Chlorogenic acid | C_16_H_18_O_9_ | -0.53 | 354.09489 | 3.533 | 353.08762 |
| 15 | N-Acetyl-DL-norvaline | C_7_H_13_NO_3_ | -0.81 | 159.08941 | 3.683 | 158.08214 |
| 16 | Pyrrole-2,5-dicarboxylic acid | C_6_H_5_NO_4_ | -0.84 | 155.02173 | 3.696 | 154.01445 |
| 17 | Lotusine | C_19_H_23_NO_3_ | -0.6 | 313.16761 | 4.283 | 312.16037 |
| 18 | Methyl gallate | C_8_H_8_O_5_ | -0.77 | 184.03703 | 4.723 | 183.02975 |
| 19 | Kynurenic acid | C_10_H_7_NO_3_ | -0.93 | 189.04242 | 4.794 | 188.03514 |
| 20 | Quercetin 3-O-β-D-glucose-7-O-β-D-gentiobioside | C_33_H_40_O_22_ | -0.77 | 788.20051 | 4.992 | 787.19324 |
| 21 | (+)-Catechin | C_15_H_14_O_6_ | -0.05 | 290.07902 | 5.031 | 289.07175 |
| 22 | Aucubin | C_15_H_22_O_9_ | -0.87 | 346.12608 | 5.145 | 391.12424 |
| 23 | D(-)-Amygdalin | C_20_H_27_NO_11_ | -1.11 | 457.1579 | 5.385 | 502.15607 |
| 24 | Albiflorin | C_23_H_28_O_11_ | -0.64 | 480.16286 | 6.799 | 479.15558 |
| 25 | 3-Coumaric acid | C_9_H_8_O_3_ | -0.45 | 164.04727 | 7.675 | 163.03999 |
| 26 | Chrysin 6-C-arabinoside 8-C-glucoside | C_26_H_28_O_13_ | -0.42 | 548.15276 | 9.326 | 547.14551 |
| 27 | Rutin | C_27_H_30_O_16_ | 0.32 | 610.15358 | 9.795 | 609.1463 |
| 28 | Isoacteoside | C_29_H_36_O_15_ | -1.02 | 624.20479 | 10.715 | 623.19751 |
| 29 | 3-Hydroxybenzoic acid | C_7_H_6_O_3_ | -0.8 | 138.03158 | 10.877 | 137.02431 |
| 30 | Tectoridin | C_22_H_22_O_11_ | -0.75 | 462.11587 | 11.059 | 461.10852 |
| 31 | NP-018732 | C_26_H_28_O_13_ | -0.26 | 548.15285 | 11.291 | 547.14557 |
| 32 | Guaijaverin | C_20_H_18_O_11_ | -0.56 | 434.08467 | 11.91 | 433.07739 |
| 33 | Verbascoside | C_29_H_36_O_15_ | -1.11 | 624.20473 | 11.946 | 623.19745 |
| 34 | 3,5-Dicaffeoylquinic acid | C_25_H_24_O_12_ | -0.93 | 516.1263 | 12.065 | 515.11902 |
| 35 | Prunin | C_21_H_22_O_10_ | -0.47 | 434.12109 | 12.177 | 415.10324 |
| 36 | DL-Arginine | C_6_H_14_N_4_O_2_ | -0.15 | 174.11165 | 12.544 | 175.11893 |
| 37 | Choline | C_5_H_13_NO | -0.26 | 103.09969 | 12.61 | 104.10696 |
| 38 | DL-Carnitine | C_7_H_15_NO_3_ | -0.17 | 161.10517 | 12.624 | 162.11244 |
| 39 | 7-Hydroxy-2,5-dimethylchromone | C_11_H_10_O_3_ | -0.44 | 190.06291 | 12.782 | 189.05563 |
| 40 | Propionylcarnitine | C_10_H_19_NO_4_ | 0.15 | 217.13144 | 12.85 | 218.13872 |
| 41 | Naringenin-4'-O-β-D-glucuronide | C_21_H_20_O_11_ | -0.32 | 448.10042 | 12.894 | 447.09314 |
| 42 | Apigenin 7-O-glucuronide | C_21_H_18_O_11_ | -0.47 | 446.0847 | 13.203 | 445.07742 |
| 43 | Lridin | C_24_H_26_O_13_ | -0.85 | 522.1369 | 13.233 | 521.1297 |
| 44 | Homoplantaginin | C_22_H_22_O_11_ | -0.04 | 462.11619 | 13.609 | 461.10892 |
| 45 | 4,5-Dicaffeoylquinic acid | C_25_H_24_O_12_ | 0.13 | 516.12684 | 13.613 | 515.11957 |
| 46 | 5-hydroxy-4-methoxy-5,6-dihydro-2H-pyran-2-one | C_6_H_8_O_4_ | -0.14 | 144.04224 | 13.626 | 145.04951 |
| 47 | Phloroglucinol | C_6_H_6_O_3_ | -0.2 | 126.03167 | 14.114 | 127.03895 |
| 48 | 5-Oxoprolylproline | C_10_H_14_N_2_O_4_ | -0.15 | 226.09532 | 14.12 | 227.1026 |
| 49 | Eriodictyol | C_15_H_12_O_6_ | 0.25 | 288.06346 | 14.646 | 287.05618 |
| 50 | Afzelin | C_21_H_20_O_10_ | -0.31 | 432.10551 | 14.91 | 431.09824 |
| 51 | Val-Leu | C_11_H_22_N_2_O_3_ | -0.13 | 230.16301 | 14.933 | 231.17029 |
| 52 | Baicalin | C_21_H_18_O_11_ | -0.88 | 446.08452 | 15.882 | 445.07724 |
| 53 | N-Acetyldopamine | C_10_H_13_NO_3_ | -0.05 | 195.08953 | 16.598 | 196.09682 |
| 54 | Epicatechin | C_15_H_14_O_6_ | 0.07 | 290.07906 | 16.867 | 291.08633 |
| 55 | Xanthurenic acid | C_10_H_7_NO_4_ | -0.3 | 205.03745 | 16.903 | 206.04472 |
| 56 | Dihydrobaicalin | C_21_H_20_O_11_ | -0.6 | 448.10029 | 17.048 | 447.09302 |
| 57 | 2-(4-Hydroxy-3-methoxyphenyl)ethyl 3-O-(6-deoxyhexopyranosyl)-4-O-[(2E)-3-(4-hydroxy-3-methoxyphenyl)-2-propenoyl]hexopyranoside | C_31_H_40_O_15_ | -0.16 | 652.23662 | 17.296 | 651.22931 |
| 58 | Quercetin | C_15_H_10_O_7_ | -0.32 | 302.04255 | 18.087 | 301.03528 |
| 59 | Isorhamnetin | C_16_H_12_O_7_ | -0.1 | 316.05827 | 18.333 | 315.05099 |
| 60 | Coclaurine | C_17_H_19_NO_3_ | 0.21 | 285.13655 | 18.517 | 286.14383 |
| 61 | (1r,3R,4s,5S)-4-{[(2E)-3-(3,4-dihydroxyphenyl)prop-2-enoyl]oxy}-1,3,5-trihydroxycyclohexane-1-carboxylic acid | C_16_H_18_O_9_ | 0.27 | 354.09518 | 18.52 | 355.10245 |
| 62 | 6-Methoxyluteolin | C_16_H_12_O_7_ | 0.19 | 316.05836 | 18.734 | 315.05109 |
| 63 | 7-Acetyl-8-hydroxy-3-methoxy-6-methyl-1-naphthyl beta-D-glucopyranoside | C_20_H_24_O_9_ | -0.72 | 408.14174 | 18.835 | 407.13446 |
| 64 | Magnoflorine | C_20_H_23_NO_4_ | -0.47 | 341.16255 | 19.123 | 342.16983 |
| 65 | Pseudolaric acid C | C_21_H_26_O_7_ | -0.45 | 390.16768 | 19.207 | 389.1604 |
| 66 | Apigenin 7-O-glucoside | C_21_H_20_O_10_ | -0.38 | 432.10548 | 19.214 | 431.09821 |
| 67 | Cirsiliol | C_17_H_14_O_7_ | -0.26 | 330.07387 | 19.365 | 329.06659 |
| 68 | Apigetrin | C_21_H_20_O_10_ | -0.52 | 432.10542 | 19.702 | 431.09814 |
| 69 | Sinomenine | C_19_H_23_NO_4_ | 0.46 | 329.16286 | 19.776 | 330.17014 |
| 70 | Wogonoside | C_22_H_20_O_11_ | -1.24 | 460.09999 | 20.661 | 459.09271 |
| 71 | Luteolin | C_15_H_10_O_6_ | -0.2 | 286.04768 | 20.405 | 285.04041 |
| 72 | Benzoylpaeoniflorin | C_30_H_32_O_12_ | -0.95 | 584.18882 | 22.274 | 629.18701 |
| 73 | Alizarin | C_14_H_8_O_4_ | -1.18 | 240.04198 | 24.075 | 239.0347 |
| 74 | Kaempferol | C_15_H_10_O_6_ | 0.12 | 286.04777 | 24.08 | 285.0405 |
| 75 | Moracin M | C_14_H_10_O_4_ | 0.11 | 242.05794 | 24.086 | 241.05066 |
| 76 | Chrysophanol | C_15_H_10_O_4_ | -0.13 | 254.05787 | 24.555 | 253.0506 |
| 77 | Scutellarin | C_21_H_18_O_12_ | 0.3 | 462.07996 | 24.577 | 463.08725 |
| 78 | Malvidin | C_17_H_14_O_7_ | 0.12 | 330.07399 | 25.471 | 331.08127 |
| 79 | Puerarin | C_21_H_20_O_9_ | 0.01 | 416.11073 | 25.821 | 417.11801 |
| 80 | NP-019498 | C_28_H_32_O_16_ | 0.72 | 624.16948 | 25.875 | 625.17676 |
| 81 | 5-hydroxy-2-(4-hydroxy-3-methoxyphenyl)-6-methoxy-7-{[(2S,3R,4S,5S,6R)-3,4,5-trihydroxy-6-(hydroxymethyl)oxan-2-yl]oxy}-4H-chromen-4-one | C_23_H_24_O_12_ | -0.08 | 492.12674 | 26.693 | 493.134 |
| 82 | 3,8-dihydroxy-1-methylanthraquinone-2-carboxylic acid | C_16_H_10_O_6_ | -0.19 | 298.04768 | 27.635 | 297.04041 |
| 83 | Questin | C_16_H_12_O_5_ | -0.46 | 284.06834 | 27.881 | 283.06107 |
| 84 | Genistin | C_21_H_20_O_10_ | -0.02 | 432.10564 | 28.854 | 433.11292 |
| 85 | Rhein | C_15_H_8_O_6_ | 0.43 | 284.03221 | 30.32 | 283.02493 |
| 86 | Hedysarimcoumestan B | C_16_H_10_O_6_ | -0.19 | 298.04768 | 30.369 | 297.04041 |
| 87 | Glychionide A | C_21_H_18_O_11_ | -0.53 | 446.08468 | 31.034 | 447.09195 |
| 88 | 5-hydroxy-2-(4-hydroxyphenyl)-6-methoxy-7-{[(2S,3R,4S,5S,6R)-3,4,5-trihydroxy-6-(hydroxymethyl)oxan-2-yl]oxy}-4H-chromen-4-one | C_22_H_22_O_11_ | 0 | 462.11621 | 31.167 | 463.1235 |
| 89 | 3-(4-hydroxyphenyl)-7-methoxy-5-{[(3R,4S,5S,6R)-3,4,5-trihydroxy-6-(hydroxymethyl)oxan-2-yl]oxy}-4H-chromen-4-one | C_22_H_22_O_10_ | 0.12 | 446.12135 | 31.65 | 447.1286 |
| 90 | 4-(5,7-Dihydroxy-4-oxo-4H-chromen-3-yl)phenyl beta-D-glucopyranosiduronic acid | C_21_H_18_O_11_ | -0.19 | 446.08483 | 31.819 | 447.0921 |
| 91 | (2S,3S,4S,5R,6S)-3,4,5-trihydroxy-6-[(5-hydroxy-8-methoxy-4-oxo-2-phenyl-4H-chromen-7-yl)oxy]oxane-2-carboxylic acid | C_22_H_20_O_11_ | -0.59 | 460.10029 | 32.052 | 461.10754 |
| 92 | Diosmetin 7-glucuronide | C_22_H_20_O_12_ | -0.32 | 476.09532 | 32.249 | 477.1026 |
| 93 | Octyl hydrogen sulfate | C_8_H_18_O_4_S | -1.19 | 210.09233 | 32.515 | 209.08505 |
| 94 | Skullcapflavone II | C_19_H_18_O_8_ | -0.97 | 374.09981 | 32.611 | 373.09253 |
| 95 | Apocynin | C_9_H_10_O_3_ | -1.31 | 166.06278 | 33.081 | 167.07005 |
| 96 | Saikosaponin B2 | C_42_H_68_O_13_ | -0.38 | 780.4657 | 33.77 | 825.46393 |
| 97 | Soyasaponin I | C_48_H_78_O_18_ | 0.26 | 942.51906 | 33.978 | 941.51166 |
| 98 | Pelargonidin | C_15_H_10_O_5_ | -0.38 | 270.05272 | 34.015 | 271.06 |
| 99 | 1,3-Dihydroxy-2,7,8-trimethoxy-6-methyl-9,10-anthracenedione | C_18_H_16_O_7_ | -0.5 | 344.08943 | 34.061 | 343.08215 |
| 100 | Saikosaponin B1 | C_42_H_68_O_13_ | 0.06 | 780.46604 | 34.534 | 825.46429 |
| 101 | 5,6,7-trihydroxy-2-(4-methoxyphenyl)-4H-chromen-4-one | C_16_H_12_O_6_ | -0.16 | 300.06334 | 34.839 | 301.07062 |
| 102 | Cinnamyl alcohol | C_9_H_10_O | -0.09 | 134.07315 | 35.209 | 135.08043 |
| 103 | Calenduloside F | C_42_H_66_O_14_ | 0.13 | 794.44536 | 35.24 | 839.4436 |
| 104 | Saikosaponin D | C_42_H_68_O_13_ | -0.54 | 780.46557 | 35.73 | 825.46381 |
| 105 | Apigenin | C_15_H_10_O_5_ | -0.5 | 270.05269 | 35.843 | 269.04538 |
| 106 | (+/-)9,10-dihydroxy-12Z-octadecenoic acid | C_18_H_34_O_4_ | 0 | 314.24571 | 36.192 | 313.23843 |
| 107 | Honokiol | C_18_H_18_O_2_ | -0.19 | 266.13063 | 36.508 | 265.12335 |
| 108 | Spiculisporic acid | C_17_H_28_O_6_ | -0.21 | 328.18852 | 37.124 | 327.18124 |
| 109 | (2S)-3-{[6-O-(alpha-D-Galactopyranosyl)-beta-D-galactopyranosyl]oxy}-2-hydroxypropyl (9Z,12Z)-9,12-octadecadienoate | C_33_H_58_O_14_ | -0.27 | 678.38248 | 37.449 | 723.38068 |
| 110 | 5,7-dihydroxy-2-(3-hydroxy-4-methoxyphenyl)-3,6-dimethoxy-4H-chromen-4-one | C_18_H_16_O_8_ | -0.25 | 360.08443 | 37.772 | 361.09171 |
| 111 | 13(S)-HOTrE | C_18_H_30_O_3_ | -0.1 | 294.21947 | 38.777 | 293.21219 |
| 112 | Corosolic acid | C_30_H_48_O_4_ | 0.05 | 472.35529 | 38.23 | 471.34799 |
| 113 | (±)12(13)-DiHOME | C_18_H_34_O_4_ | 0.2 | 314.24577 | 39.139 | 313.23849 |
| 114 | Ursolic acid | C_30_H_48_O_3_ | -0.11 | 456.36029 | 41.643 | 455.35303 |
| 115 | 3,3',4'-TRIMETHOXYFLAVONE | C_18_H_16_O_5_ | 0.02 | 312.09978 | 42.401 | 313.10706 |
| 116 | Lauryldimethylamine oxide | C_14_H_31_NO | -0.23 | 229.24051 | 42.837 | 230.24779 |
| 117 | Irisflorentin | C_20_H_18_O_8_ | -0.45 | 386.09999 | 44.13 | 387.10724 |
| 118 | 12-Hydroxy-14-methoxy-3-methyl-3,4,5,6,7,8,9,10-octahydro-1H-2-benzoxacyclododecin-1-one | C_17_H_24_O_4_ | 0.24 | 292.16753 | 48.33 | 293.1748 |
| 119 | Glycitein | C_16_H_12_O_5_ | -0.88 | 284.06822 | 44.912 | 285.0755 |
| 120 | Chrysin | C_15_H_10_O_4_ | -0.12 | 254.05788 | 45.342 | 255.06516 |
| 121 | Wogonin | C_16_H_12_O_5_ | -0.45 | 284.06835 | 46.035 | 285.07562 |
| 122 | 4-Ethoxy ethylbenzoate | C_11_H_14_O_3_ | -0.12 | 194.09427 | 47.956 | 195.10155 |
| 123 | 3-Methoxybenzaldehyde | C_8_H_8_O_2_ | -0.21 | 136.0524 | 49.378 | 137.05968 |
| 124 | Asperphenamate_120258 | C_32_H_30_N_2_O_4_ | 0.17 | 506.22064 | 49.726 | 507.22791 |
| 125 | 9-Oxo-ODE | C_18_H_30_O_3_ | 0.02 | 294.2195 | 50.654 | 295.22678 |
| 126 | 9-Oxo-10(E),12(E)-octadecadienoic acid | C_18_H_30_O_3_ | 0 | 294.2195 | 51.005 | 295.22678 |
| 127 | Octyl hydrogen phthalate | C_16_H_22_O_4_ | 0 | 278.15181 | 51.142 | 279.15909 |
| 128 | Oleoyl ethanolamide | C_20_H_39_NO_2_ | 0.18 | 325.29814 | 53.368 | 326.30542 |
| 129 | Hexadecanamide | C_16_H_33_NO | -0.25 | 255.25615 | 53.685 | 256.26343 |
| 130 | Oleamide | C_18_H_35_NO | 0.12 | 281.2719 | 54.485 | 282.27917 |
| 131 | Erucamide | C_22_H_43_NO | 0.25 | 337.33455 | 55.951 | 338.34183 |
| 132 | Stearamide | C_18_H_37_NO | 0.14 | 283.28755 | 56.291 | 284.29483 |
| 133 | Diisooctyl phthalate | C_24_H_38_O_4_ | 0.28 | 390.27712 | 59.747 | 413.26633 |

***Supplementary Figure Legends***

**Supplementary Fig. S1 Constructing the regulatory network of BJJP against HF.**

(A)Visualization network diagram of core active compounds of BJJP-key targets-disease pathways. Among them, arrows represent characteristic genes, parallelograms represent pathways, and triangles represent compounds. (B) Diagram of miRNA-TF-mRNA regulatory network, where the arrows represent characteristic genes, the hexagons represent miRNA, and the ellipses represent TF. TF:Transcription factor.

**Supplementary Fig. S2 Analysis of immune infiltration in HF by ssGSEA .**

(A)Visualization of the proportion distribution of immune cell subsets in the control and HF groups. (B) Heatmap with hierarchical clustering of immune cell subsets in the control and HF groups. (C) Box plot of the proportion distribution of immune cell subsets in the control and HF groups. ^*^*P*< 0.05, ^**^*P*<0.01,^***^*P*< 0.001, ns:no significant.

**Supplementary Fig. S3 The identification result of the chemical compounds of BJJP by UHPLC-HRMS.**

(A)The positive-mode total ion current chromatogram of BJJP. (B) The negative-mode total ion current chromatogram of BJJP. (C) The 254 nm chromatogram of BJJP.

***Supplementary Figures***

**Supplementary Fig. S1**

**
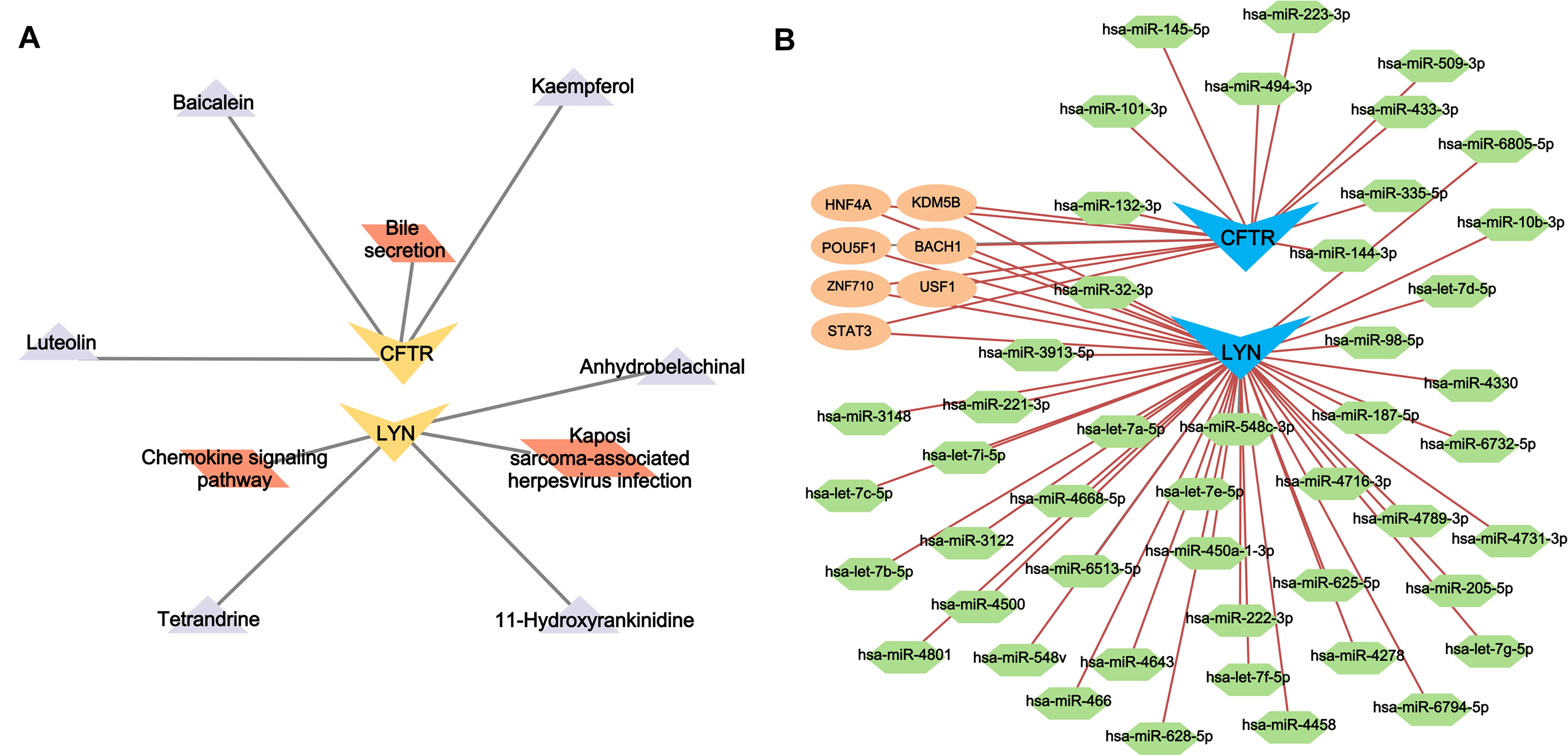
**

**Supplementary Fig. S2**

**
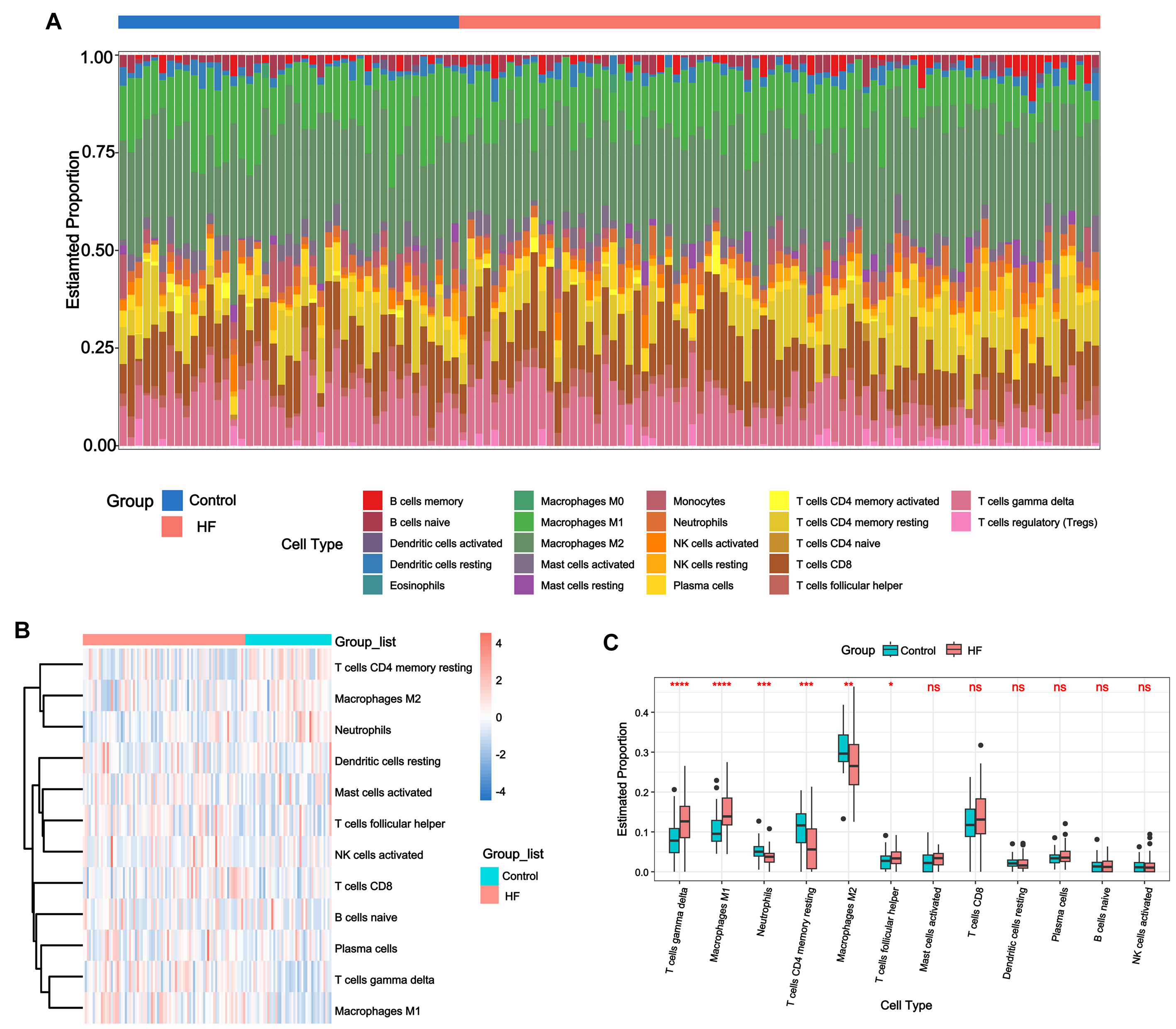
**

**Supplementary Fig. S3**

**
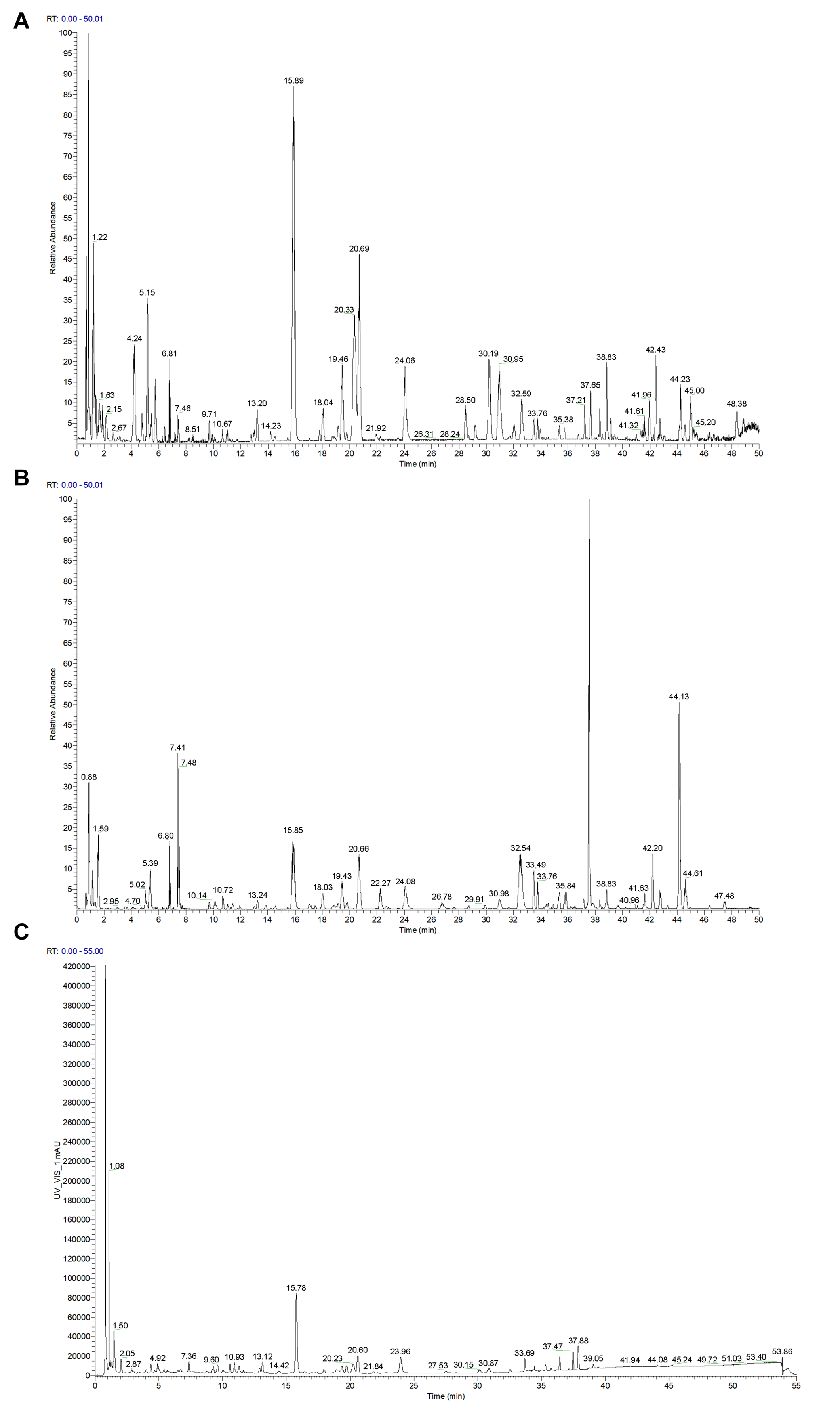
**

**REFERENCES**

Atwell, S., Brouillette, C. G., Conners, K., Emtage, S., Gheyi, T., Guggino, W. B.,... Zhao, X. (2010). Structures of a minimal human CFTR first nucleotide-binding domain as a monomer, head-to-tail homodimer, and pathogenic mutant. *Protein Engineering Design & Selection*, *23* (5), 375-384. http://doi.org/10.1093/protein/gzq004.

Berndt, S., Gurevich, V. V., & Iverson, T. M. (2019). Crystal structure of the SH3 domain of human Lyn non-receptor tyrosine kinase. *Plos One*, *14* (4), e215140. http://doi.org/10.1371/journal.pone.0215140.

Mceligot, A. J., Poynor, V., Sharma, R., & Panangadan, A. (2020). Logistic LASSO Regression for Dietary Intakes and Breast Cancer. *Nutrients*, *12* (9) http://doi.org/10.3390/nu12092652.

Reimand, J., Isserlin, R., Voisin, V., Kucera, M., Tannus-Lopes, C., Rostamianfar, A.,... Bader, G. D. (2019). Pathway enrichment analysis and visualization of omics data using g:Profiler, GSEA, Cytoscape and EnrichmentMap. *Nature Protocols*, *14* (2), 482-517. http://doi.org/10.1038/s41596-018-0103-9.

Sanz, H., Valim, C., Vegas, E., Oller, J. M., & Reverter, F. (2018). SVM-RFE: selection and visualization of the most relevant features through non-linear kernels. *Bmc Bioinformatics*, *19* (1), 432. http://doi.org/10.1186/s12859-018-2451-4.

Utkin, L. V., & Konstantinov, A. V. (2022). Attention-based random forest and contamination model. *Neural Networks*, *154*, 346-359. http://doi.org/10.1016/j.neunet.2022.07.029.

Yu, G., Wang, L. G., Han, Y., & He, Q. Y. (2012). clusterProfiler: an R package for comparing biological themes among gene clusters. *Omics-a Journal of Integrative Biology*, *16* (5), 284-287. http://doi.org/10.1089/omi.2011.0118.
